# The molecular basis of differential host responses to avian influenza viruses in avian species with differing susceptibility

**DOI:** 10.3389/fcimb.2023.1067993

**Published:** 2023-02-28

**Authors:** Katrina M. Morris, Anamika Mishra, Ashwin A. Raut, Eleanor R. Gaunt, Dominika Borowska, Richard I. Kuo, Bo Wang, Periyasamy Vijayakumar, Santhalembi Chingtham, Rupam Dutta, Kenneth Baillie, Paul Digard, Lonneke Vervelde, David W. Burt, Jacqueline Smith

**Affiliations:** ^1^ The Roslin Institute and R(D)SVS, The University of Edinburgh, Edinburgh, United Kingdom; ^2^ National Institute of High Security Animal Diseases, Indian Council of Agricultural Research, Bhopal, India

**Keywords:** avian influenza, transcriptome, H5N1, chicken, duck, pigeon, crow, disease resistance

## Abstract

**Introduction:**

Highly pathogenic avian influenza (HPAI) viruses, such as H5N1, continue to pose a serious threat to animal agriculture, wildlife and to public health. Controlling and mitigating this disease in domestic birds requires a better understanding of what makes some species highly susceptible (such as turkey and chicken) while others are highly resistant (such as pigeon and goose). Susceptibility to H5N1 varies both with species and strain; for example, species that are tolerant of most H5N1 strains, such as crows and ducks, have shown high mortality to emerging strains in recent years. Therefore, in this study we aimed to examine and compare the response of these six species, to low pathogenic avian influenza (H9N2) and two strains of H5N1 with differing virulence (clade 2.2 and clade 2.3.2.1) to determine how susceptible and tolerant species respond to HPAI challenge.

**Methods:**

Birds were challenged in infection trials and samples (brain, ileum and lung) were collected at three time points post infection. The transcriptomic response of birds was examined using a comparative approach, revealing several important discoveries.

**Results:**

We found that susceptible birds had high viral loads and strong neuro-inflammatory response in the brain, which may explain the neurological symptoms and high mortality rates exhibited following H5N1 infection. We discovered differential regulation of genes associated with nerve function in the lung and ileum, with stronger differential regulation in resistant species. This has intriguing implications for the transmission of the virus to the central nervous system (CNS) and may also indicate neuro-immune involvement at the mucosal surfaces. Additionally, we identified delayed timing of the immune response in ducks and crows following infection with the more deadly H5N1 strain, which may account for the higher mortality in these species caused by this strain. Lastly, we identified candidate genes with potential roles in susceptibility/resistance which provide excellent targets for future research.

**Discussion:**

This study has helped elucidate the responses underlying susceptibility to H5N1 influenza in avian species, which will be critical in developing sustainable strategies for future control of HPAI in domestic poultry.

## 1 Introduction

Avian influenza, caused by highly pathogenic avian influenza viruses (HPAI) such as H5N1, is responsible for enormous economic losses in the poultry industry, causes devastating effects of wildlife and poses a serious pandemic threat to public health. To date, H5N1 has affected the poultry industry in 68 countries, with over 15,000 outbreaks reported, and has become endemic in six countries (Bangladesh, China, Egypt, India, Indonesia and Vietnam; Alexander and Brown, 2009; Bui et al., 2016). Avian influenza has a particularly devastating impact in developing countries, where outbreaks have a huge social and economic impact on small and marginal poultry farmers (Rushton et al., 2005; Brown, 2010; Alders et al., 2014). In addition it has devastating impacts on wild bird populations and poses a serious conservation threat (Daszak et al., 2000), highlighted by recent widespread outbreaks in wild bird populations (at least 57 species) in Europe (EFSA (European Food Safety Authority), 2022)

Due to the close proximity of domestic species, wild birds and humans in backyard farms and bird markets in countries such as India and China, the risk of novel zoonoses is high and the fear of the emergence of a devastating pandemic flu ever-present. Since the emergence of H5N1 in 1997, there has been an increasing number of bird-to-human transmission events (WHO (World Health Organisation), 2021a). Over 800 cases of H5N1 infections in humans have been confirmed across 17 countries as of 2021, with a mortality rate of over 50% in those infected (WHO (World Health Organisation), 2021a). Previous influenza pandemics are estimated to have collectively killed 25 to over 100 million people (WHO (World Health Organisation), 2013; Spreeuwenberg et al., 2018). It is essential that the spread of H5N1 be controlled, particularly in domestic birds, where the risk of transmission to humans is the highest. The recent COVID19 pandemic has highlighted the devastating economic and human impact such zoonotic diseases pose (WHO (World Health Organisation), 2021b). Control of this disease in domestic birds is therefore critical for food biosecurity, animal welfare and public health.

H5N1 is known to infect a variety of both domestic and wild avian species; however, the response to infection varies widely. Chickens typically exhibit high mortality in outbreaks of H5N1, with case fatality rates as high as 100% (Kayali et al., 2011). Ducks and other waterfowl are generally asymptomatic and can act as reservoirs of H5N1 (Walsh et al., 2017). Additionally, wild species such as pigeons and crows also appear to be generally resistant to the effects of H5N1 (Yamamoto et al., 2012). Strikingly in a 2011 outbreak in India, high mortality from H5N1 was observed in the normally H5N1-resistant crows and ducks (Khan et al., 2014). The mechanisms that promote high pathogenicity of these recent clades of HPAI are not understood. Though the pathogenesis of HPAI in chickens and ducks is known to some extent, little is understood about mechanisms of resistance in other birds, which can act as reservoirs of infection to poultry. Resistant species may have mechanisms able to prevent viral replication and spread, check disease progression or mitigate immunopathology.

Despite measures to control the disease, HPAI continues to be detected globally in wild birds, even in the absence of local poultry outbreaks (Walsh et al., 2017). At present HPAI outbreaks among poultry are usually controlled by draconian measures, with the culling of all susceptible commercial flocks in affected areas and areas at risk (Alexander and Brown, 2009). Vaccination has not had widespread success in controlling the disease, partly due to the rapid evolution of influenza viruses and insufficient cross-protection between different variants (Kayali et al., 2013; Spackman and Swayne, 2013; Kim, 2018). As HPAI can be spread by free-flying species (Walsh et al., 2017) which cannot be contained, new approaches to HPAI control are needed.

The overall aim of this study was to understand the genetic differences in the host that set the balance of disease resistance versus susceptibility for individual avian host-pathogen combinations. In order to examine the differences in the molecular signatures of hosts differing in their susceptibility to avian influenza we took a comparative transcriptomic approach. Six avian species - chickens/turkeys (highly susceptible with high mortality), geese/pigeons (tolerant carriers with only sporadic mortality) and ducks/crows (resistant to most AIV infections but having differential responses to virus of different H5 clades) were infected with two H5N1 strains from different clades, as well as low pathogenic (LPAI) H9N2 virus. The knowledge gained from these comparisons can be used to develop sustainable strategies to control HPAI infections in domestic poultry.

## 2 Materials and methods

### 2.1 Virus strains

The three wild-type virus strains used for infections were H9N2 (A/duck/India/249800/2010) hereafter referred to as H9, H5N1 clade 2.2 (A/duck/Tripura/103597/2008) hereafter referred to as 2.2 and H5N1 clade 2.3.2.1 (A/duck/India/02CA10/2011) hereafter referred to as 2.3. All viruses were grown on 11-day-old embryonated duck eggs of white Pekin ducks at ICAR-NIHSAD (Bhopal, India). The experimental birds were infected intra-nasally with 106 EID50 of the virus in 100µl of allantoic fluid, with volume adjusted with PBS.

### 2.2 Animals

All animal experiments were approved by the ICAR institute animal ethics committee (IAEC) approval (73/IAEC/HSADL/13). Six species were examined in three groups. Group 1 - Chicken (White leghorn, Gallus gallus domesticus) and turkey (Beltsville, Meleagris gallopavo), Group 2 - mallard duck (Pekin duck, Anas platyrhnchos domesticus) and jungle crow (Corvus macrorhynchos) and Group 3 - greylag goose (Embden goose, Anser anser domesticus) and rock dove (pigeon, Columba livia). Chickens and turkeys were procured from the Central Poultry Development Organization, Mumbai, India as day old chicks and reared for 3 weeks in a well-drained concrete floored animal house at the receiving shed and for the next 3-5 weeks in a holding shed at NIHSAD, Bhopal. The ducks were procured from the Nature and Culture Society, Malur, India, as day old ducklings and reared in wire mesh floored, positive pressure isolators up to 6-8 weeks. Geese (11-13 months old), crows (age not known) and pigeons (age not known) were procured from the local live bird market in Bhopal, India and housed for 3 weeks in a receiving shed to meet institutional quarantine requirements. Sexing was done by PCR and only females were included in the study. Haemagglutination inhibition tests were used to screen the birds to confirm that they were free from AIV antibodies, using the viruses H9N2 (A/duck/India/249800/2010), H5N1 (A/duck/Tripura/103597/2008) and H5N1 (A/duck/India/02CA10/2011).

### 2.3 Preliminary trials

A preliminary infection course trial was carried out in crows, pigeons and geese to ensure that the AIV of duck origin were able to infect these species. The purpose here was also to observe the course of disease in these species to determine the appropriate time point for sampling of infected birds for transcriptome analysis. For each species, 3 groups of 6 birds were experimentally challenged intra-nasally with each of the three viruses at a dose of 106 EID50 virus in 100 μl allantoic fluid diluted in PBS. The birds were observed for clinical signs and oral as well as cloacal swabs were collected at 24-48 hr intervals up to 10 days.

### 2.4 Infection trials

For the challenge experiments, 5 birds were infected for each treatment group, to ensure sufficient sample numbers for RNA-seq, the birds of each species were grouped into 3 groups (A, B and C) of 15 birds each and a fourth control group (D) of 5 birds. The birds from Group A were intra-nasally infected with 10^6^ EID_50_ of 2.3. The birds from Group B were intra-nasally infected with 10^6^ EID_50_ of 2.2. The birds from Group C were intra-nasally infected with 10^6^ EID_50_ of H9. Group D was inoculated with PBS intra-nasally. The control birds were euthanised 48 hours after inoculation. In each group A-C, 5 birds were euthanised by cervical dislocation at each time point as outlined per species and infection in **Table 1**. No early deaths of experimental animals occurred. Times of tissue collection post infection differ between some groups for practical reasons, for example chickens and turkeys do not generally survive an HPAI infection beyond 3 days (**Table 1**). All challenge experiments were carried out in a BSL3 facility and post-mortem collection of tissue was done in a BSL3 laboratory at ICAR-NIHSAD (India).

**Table 1:** Time points for sampling.

	H9N2	H5N1 2.2	H5N1 2.3.2.1
Chicken/Turkey	12h, 48h, 5 days	12h, 24h, 48h	12h, 24h, 48h
Duck/Crow	12h, 48h, 5 days	12h, 48h, 5 days	12h, 48h, 5 days
Goose/Pigeon	12h, 48h, 5 days	12h, 48h, 5 days	12h, 48h, 5 days

### 2.5 Tissue collection and RNA preparation

Tissues collected for RNA-seq included lung (3-5 mm cross-sections of the central to lower part of lung), ileum (1.5 cm long mid-part of ileum sectioned into three parts of mm each) and brain (3-5 mm transverse sections from the middle of the either cerebral lobe) which were stored in Trizol at -20°C until required. RNA extractions were performed using the RNeasy kit (QIAGEN). RNA quality and quantity were checked using NanoDrop (Thermofisher) and Bioanalyser (Agilent), and the three highest quality samples were selected for sequencing. Due to quality and/or quantity issues with some of the samples, 3 samples could not be obtained for sequencing for all conditions. For reasons unknown all turkey lung samples were of insufficient quality for sequencing and so were not included in any further analysis. In addition, as disparity in sample quality can affect results, samples were required to range no more than 2.5 RIN within each species × tissue group. A table showing the number of samples successfully sequenced and included in downstream analysis in each group is shown in **Supplementary File 1**. In total, 497 samples were used in the analysis.

### 2.6 RNA-sequencing and processing

For sequencing, mRNA libraries were prepared and sequenced on an Illumina Hiseq2500 platform. 36 cycles of pair-end sequencing was run generating 50 - 230 million reads per sample. Reads were quality checked using FastQC (version 0.11.2) and trimmed for quality using cutadapt (Martin, 2011). Reads were mapped to transcript sequence files from the relevant genome using Kallisto (Bray et al., 2016). As the genomes for the greylag goose and jungle crow were not available, the genomes of the closely related swan goose (Anser cygnoides) and American crow (Corvus brachyrhynchos) were used. Transcript files were obtained from NCBI as follows: Chicken (ftp://ftp.ncbi.nlm.nih.gov/genomes/Gallus_gallus/RNA/rna.fa.gz), turkey (ftp://ftp.ncbi.nlm.nih.gov/genomes/Meleagris_gallopavo/RNA/rna.fa.gz), duck (ftp://ftp.ncbi.nlm.nih.gov/genomes/all/GCF/000/971/095/GCF_000971095.1_AnsCyg_PRJNA183603_v1.0/), crow (ftp://ftp.ncbi.nlm.nih.gov/genomes/Corvus_brachyrhynchos/RNA/rna.fa.gz), goose (ftp://ftp.ncbi.nlm.nih.gov/genomes/all/GCF/000/971/095/GCF_000971095.1_AnsCyg_PRJNA183603_v1.0/) and pigeon (ftp://ftp.ncbi.nlm.nih.gov/genomes/Columba_livia/RNA/rna.fa.gz). To allow for comparison of genes between species, the transcript level abundance data were converted to gene level data with tximport in R (Soneson et al., 2015). Differential expression analysis was performed in EdgeR using a glm model (Robinson et al., 2010). The cut-offs for significance were FDR < 0.05 and logFC > 1.5. For most cross-species analyses, a set of 11,384 genes with conserved 1-1 orthology between all six species was used. This list was generated through reciprocal BLAST searches of the genes of each species against all chicken genes, and then cross-referencing each list to produce a single list of genes conserved across all six species.

### 2.7 Data analysis

Heatmaps were constructed in R using the pheatmap package (v. 1.0.10; https://CRAN.R-project.org/package=pheatmap). Over-representation of gene ontology (GO) terms was investigated using the PANTHER Over-representation Test using Fisher’s Exact with FDR multiple test correction (Thomas et al., 2003). Network analysis for both sample-sample networks and gene-gene networks was performed in BioLayout 3D (Theocharidis et al., 2009) which performs a Pearson correlation matrix calculated for each pair of samples or genes, using a modified Fruchterman-Rheingold algorithm. Clustering was performed on these networks using the Markov clustering algorithm (MCL) with an inflation value of 1.8. The Ingenuity Pathway Analysis (IPA^®^) program (QIAGEN; Krämer et al., 2014) was used to identify cellular canonical pathways and physiological functions (p-value ≤ 0.05 and q-value ≤ 0.05). Statistical tests on RNA-seq viral counts were implemented in R 4.0.5. ANOVAs with Type III Sum of Squares were performed and Tukey Honest Significant Differences (HSD) test was used for performing multiple pairwise-comparison between the means of groups. The Tukey HSD produces p-values adjusted for the multiple comparisons.

### 2.8 Quantitative reverse transcription polymerase chain reaction

For RT-qPCR for viral genes, cDNA production and RT-qPCR were performed using the SensiFAST SYBR Lo ROX One Step kit (Bioline) as previously described (Gaunt et al., 2016). Primers targeted avian influenza A virus segment 2 (Fw 5’-CAAATACCAGCAGAAATGCTTGC; Rev 5’ TTGAACATGCCCATCATCATTCC) or avian GAPDH (Fw 5’ – TGGCCAAGGTCATCCATGACAA; Rev 5’ GATGGCATGGACAGTGGTCATAA). Reaction contained 0.8ul of 10uM of each primer. RNA samples were diluted 1 in 10 in nuclease free water and made a tenth of the total reaction volume. Amplification was performed on the Rotorgene (QIAGEN) with the following cycle conditions: 45C for 10 min, 95°C for 2 min, then 40 cycles of 95°C for 10 sec and 60°C for 30 sec, with a final melt step of 50-99°C with a 1 degree increment. Relative segment 2 expression levels were calculated using the ΔΔCt method.

For qPCR on chicken innate genes, a highly multiplexed qPCR 96.96 Fluidigm Dynamic Array system was used as described previously (Borowska et al., 2019). In brief, the Reverse transcription was performed using the High-Capacity Reverse Transcription Kit (Applied Biosystems) according to manufacturer’s instructions with random hexamers and oligo (dT)18 in a final volume of 10 μl, containing 500 ng total RNA. Pre-amplification of cDNA was performed using TaqMan PreAmp Master Mix (Applied Biosystems). Quantitative PCR was performed in the BioMark HD instrument and the 96.96 Dynamic Array (Fluidigm). Assay mixes were prepared by mixing 2.5 μl 2X Assay Loading Reagent (Fluidigm), 2.3 μl of primer pair mix (final concentration 1.15 μM) and 0.2 μl low EDTA TE buffer. Sample mixes were prepared by mixing 2.5 μl TaqMan Gene Expression Master Mix (Applied Biosystems), 0.25 μl 20X DNA Binding Dye Sample Loading Reagent (Fluidigm), 0.25 μl 20X EvaGreen DNA binding dye (Biotum) and 2 μl of preamplified cDNA. Thermal cycling conditions for qPCR were: thermal mix 50°C for 2 min, 70°C for 30 min, 25°C for 10 min, followed by hot start 50°C for 2 min, 95°C for 10 min, PCR (x30 cycles) 95°C for 15 sec, 60°C for 60 sec and melting curve analysis 60°C for 3 sec to 95°C. Real-Time PCR Analysis software 3.1.3 (Fluidigm) was used to visualise results. Analysis settings were as follows: quality threshold was set to 0.65, baseline correction to linear (derivative) and quantitation cycle (Cq) threshold method to auto (global). PCR data pre-processing, normalisation, relative quantification and statistics were performed in GenEx version 6 (MultiD Analyses AB). Data were validated and genes with >50% of missing values were removed. Data were corrected for reaction efficiency and normalised using two most stably expressed reference genes selected by NormFinder - TBP and GUSB. The dataset technical duplicates were averaged and further normalisation to control sample for each tissue was performed. Relative quantities were transformed to logarithmic scale (log2) before further analysis. Software tool for versatile matrix visualization and analysis, Morpheus by Broad Institute (RRID : SCR_017386), was used to create the gene expression heat map. All primers used for qPCR are found in **Supplementary File 2**.

## 3 Results

### 3.1 Trials

Preliminary trials were conducted to examine clinical symptoms, mortality and viral isolation in the crow, pigeon and goose over a 10 day testing period (**Supplementary File 3**). Crows and pigeons showed clinical symptoms such as ocular swelling, reduced appetite and dullness to both 2.2 and 2.3 infection after 2-5 days post infection (dpi). Goose showed no symptoms following either challenge. The only death recorded was one crow at 7 dpi after 2.3 challenge. For the goose, all tissues were positive for both 2.2 and 2.3 virus by 2 dpi. For the crow, all samples in the 2.2 challenge were positive by 2 dpi but only lung was positive for 2.3 by 2 dpi. For pigeon, all tissues were negative for virus at 2 dpi for both 2.2 and 2.3 but all samples were positive by 10 dpi. In the full animal trials, chickens and turkeys infected with both strains of H5N1 showed dullness after 24h, were off feed, and showed swollen eyes and wattles at approximately 48h. No deaths occurred during the trial period of 2-5 days.

### 3.2 Viral loads

**Figure 1:**
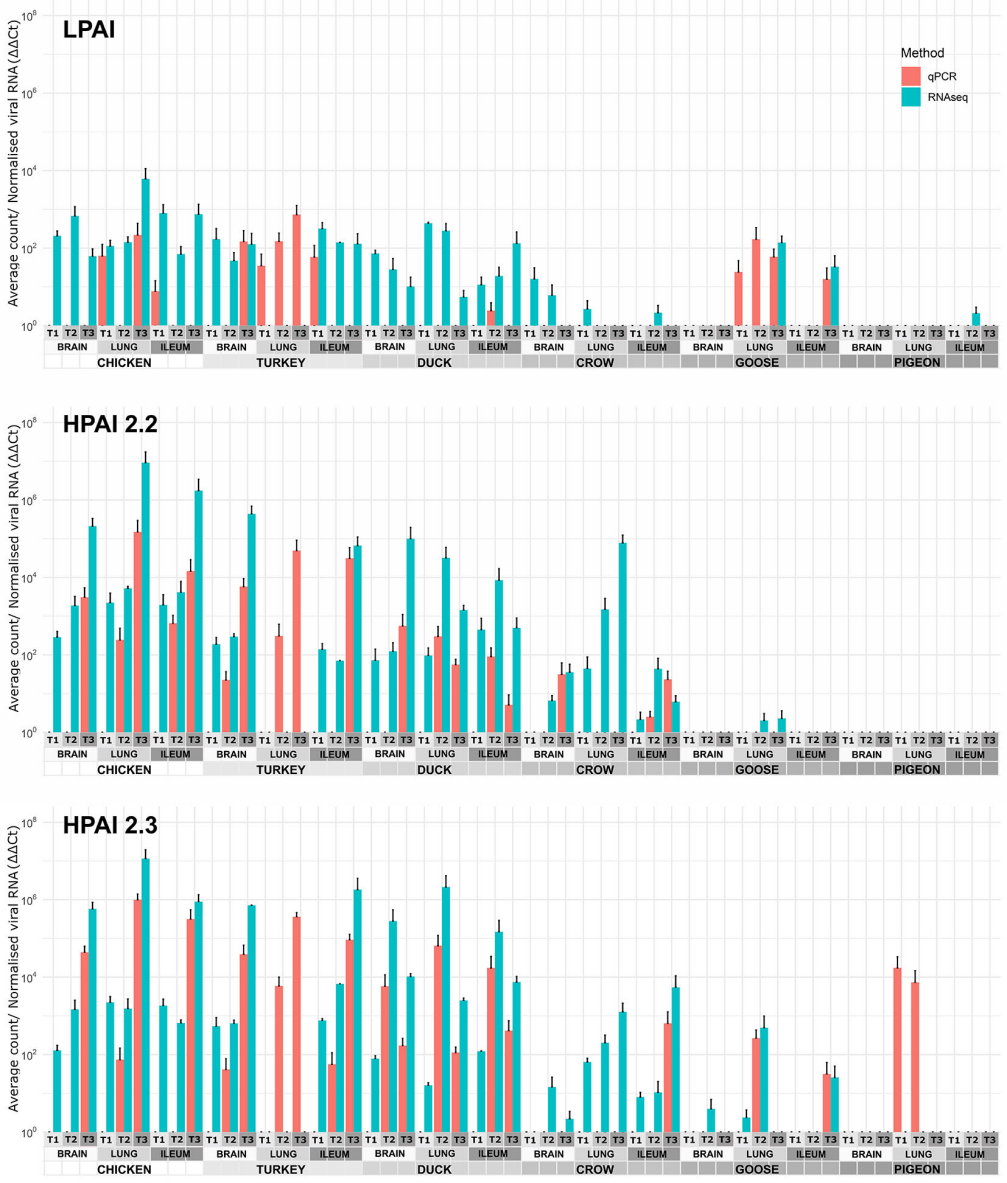
Expression of viral genes after challenge with standard error bars. Expression determined by two methods, RT-qPCR of viral genes and viral counts from RNA-seq data. Only RT-qPCR data is available for turkey lung samples. For treatment group sample sizes see **Supplementary File 1**.

The viral RNA load in each sample was determined by two methods: normalised counts of influenza viral genes from RNA-seq data (pooled) and RT-qPCR of viral genes in order to verify the RNA-seq results (**Figure 1**). In general, the two methods agreed, although the RNA-seq was more sensitive detecting viral RNA in more samples. On the other hand the RT-qPCR was able to detect viral genes in a few samples that were too degraded for successful RNA-seq, including the turkey lung samples. It is likely that the RNA-seq approach is more sensitive in higher quality samples due to high throughput, while the RT-qPCR technique was better at detecting gene expression in degraded samples as only short amplicons are amplified (145bp). As the two methods were in close agreement in samples with moderate to high levels of viral expression, both can be considered reliable, while at lower levels of expression the possibility of false negatives due to low levels of viral gene expression or sample degradation should be considered.

After H9 challenge all chicken, turkey and duck samples were positive for viral transcripts, although expression was low. Expression of viral RNA was detected in the brain of all three of these species providing evidence of systemic spread to the central nervous system even with LPAI. In crow, goose and pigeon most samples were negative for H9 with only a few samples, mostly lung samples, showing low counts in each species.

For both 2.2 and 2.3 challenges, chicken and turkey had the highest viral loads (**Figure 1**). This was statistically significant for all time points when compared to crow, goose and pigeon and most time points to duck (**Supplementary File 4**). They showed high loads in all three tissues which increased with dpi. For duck, viral loads were also high for both challenges in all tissues, but were significantly lower than turkey and chicken in the 2.2 challenge at 48h (**Supplementary File 4**). Crow had significantly lower viral loads than duck at the majority of time points, with counts being highest in lung for 2.2 and for both lung and ileum in 2.3. Goose showed very low viral load for both infections, with only viral RNA detected in the lung after 2.2 challenge, but in all three tissues after 2.3. Lastly, no viral RNA was detected in the pigeon tissues after challenge with 2.2, but was detected in lung after 2.3 infection.

### 3.3 Overviews of host responses

**Figure 2:**
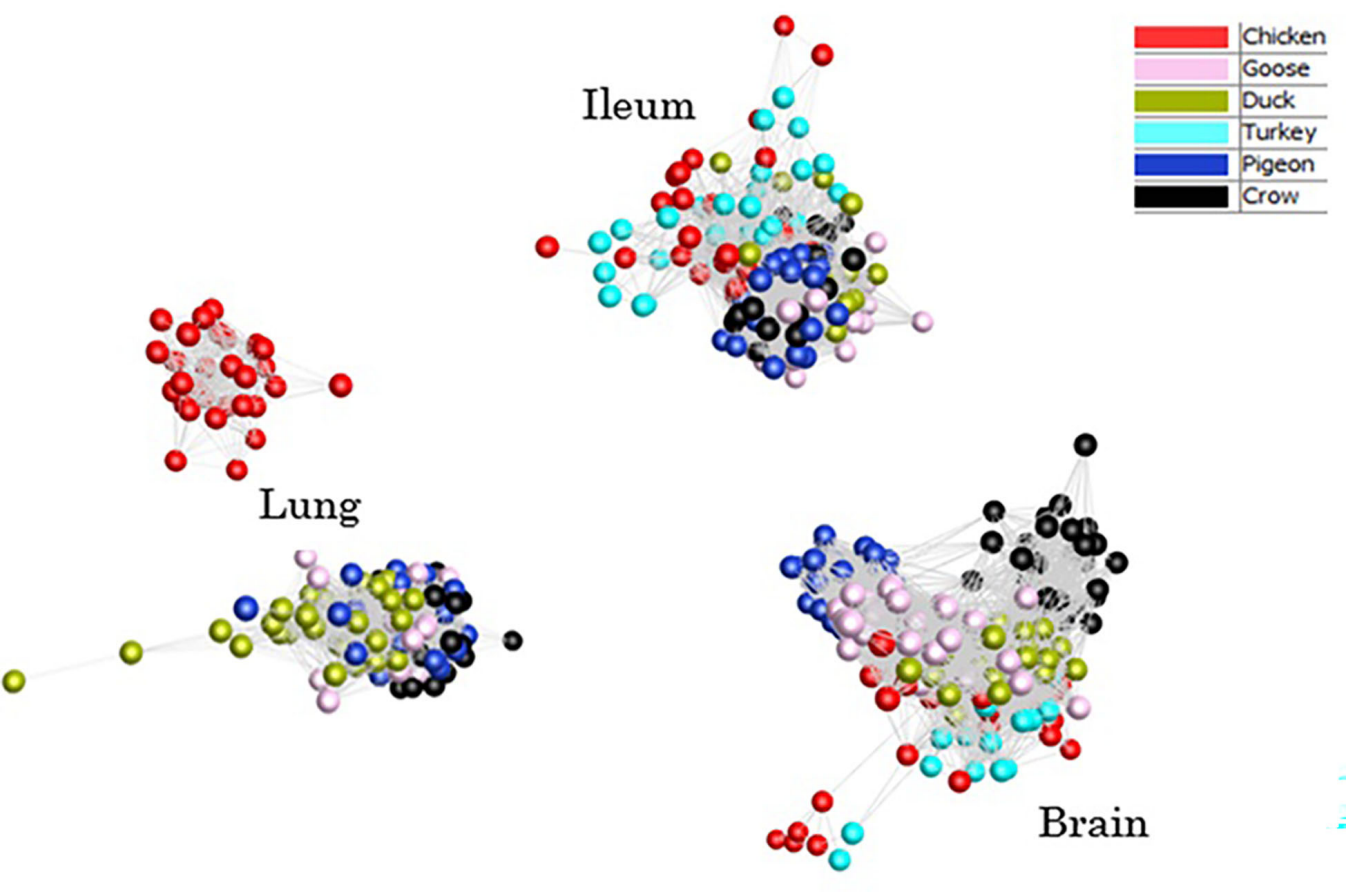
Sample-sample network graph of each individual sample normalised counts. Clustering based on Pearson correlations of gene expression in each sample. Colours are based on species with and time points are pooled.

To provide an overview of the similarity in response between all host samples a clustered sample-sample network graph was constructed (**Figure 2**), which groups samples based on similarity of gene expression. As expected, the samples clustered primarily on tissue type, with lung, ileum and brain samples clustering separately. Within each tissue, samples clustered broadly by species, although chicken and turkey samples were generally interspersed. Lung was the only tissue that did not form a single cluster – chicken lung samples clustered separately indicating a possible distinctiveness in lung gene expression and/or response to infection in this tissue. Based on the close clustering of chicken and turkey brain and ileum samples we can speculate that turkey lung samples would likely group with the chicken cluster, if present. Within each tissue cluster, the samples that formed the fewest edges with the remaining samples, which were those that were the most outlying in each cluster, were those samples from the later time points in susceptible species challenged with 2.2 and 2.3 HPAI. For example, two duck samples that lay furthest from the lung sample cluster were 2.3 infected samples from 48h and 5d. Those showing the greatest divergence from ileum and brain clusters were the 2.2 and 2.3 infected chicken and turkey samples from the 48h time-point. This may be the result of extreme or dysfunctional immune response, aberrant gene expression and/or cell death in the latter stage of disease progression.

**Figure 3:**
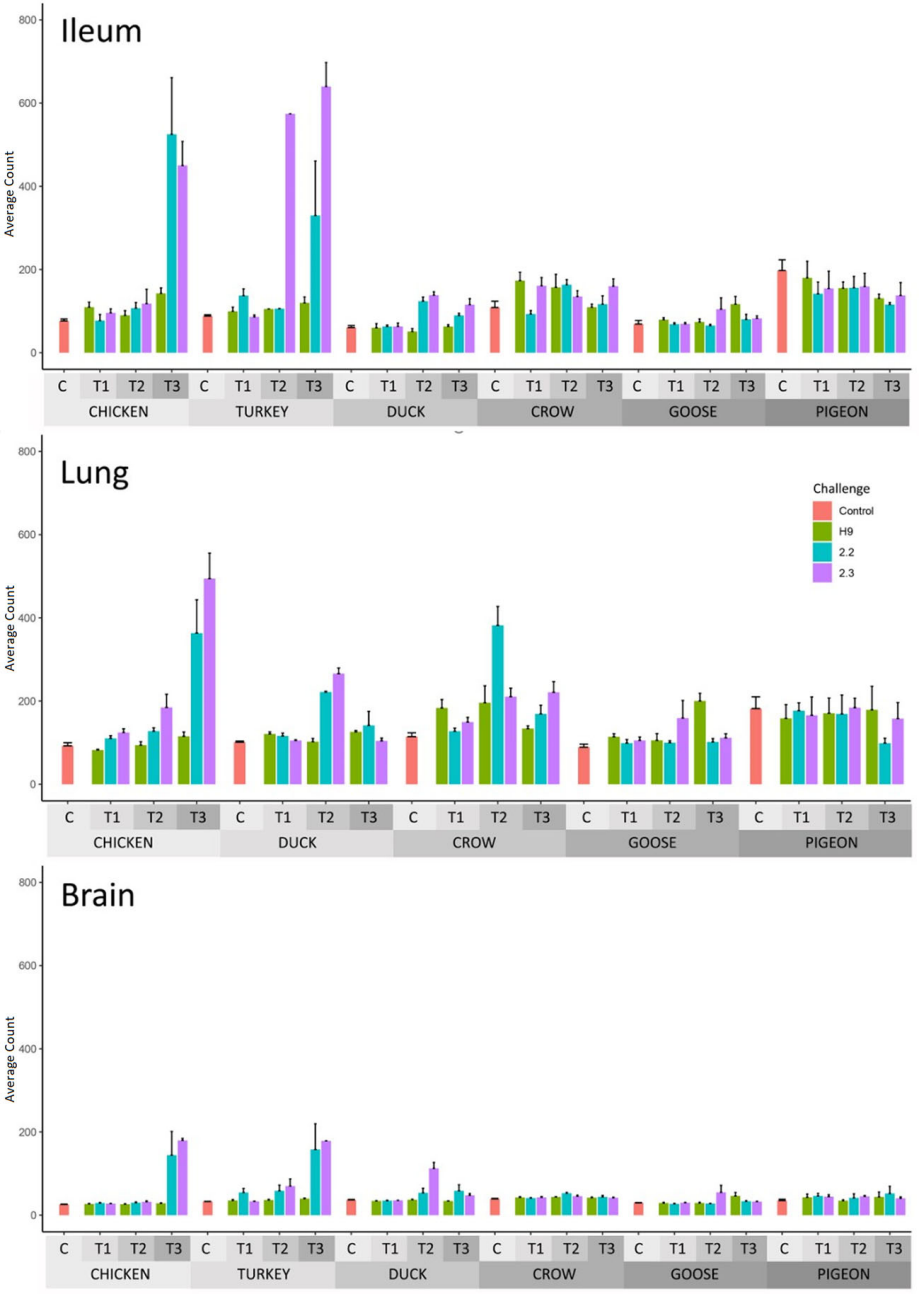
Average RNAseq read count expression of ISGs in each treatment group with Standard Error bars. Time 1, 2, 3 = 12h, 24h and 48h in chicken and turkey to 2.2 and 2.3 infection. Time 1, 2, 3 = 12h, 48h and 5d in the remaining treatment groups. For treatment group sample sizes see **Supplementary File 1**.

The number of differentially expressed genes (DEGs) for each group relative to PBS controls, is shown in **Table 2** (Full list in **Supplementary File 5**) and GO terms associated with the DEGs in **Supplementary File 6**. DEGs were identified for most groups. In most cases, the number of DEGs correlated with viral RNA load, with chicken, turkey and duck having the highest number of DEGs while crow, goose and pigeon showed lower numbers of DEGs. Expression of interferon stimulated genes (ISG) from RNA-seq data was also measured (**Figure 3**; **Supplementary File 7**) and likewise ISG expression significantly correlated with viral load (*p* < 0.01). For H9 LPAI challenge, despite all birds surviving this challenge with no clinical signs, many DEGs were identified. The number of DEGs was higher after 2.2 and 2.3 HPAI than LPAI challenge in all species. Pigeon showed the lowest number of DEGs in most tissues and time-points. However, pigeon and goose showed differential expression in all tissues, demonstrating a response to infection despite the low viral loads. The highest number of DEGs was seen in the chicken and turkey in response to 2.2 and 2.3 challenge, particularly at the later time points. The tissues showing the highest number of DEGs varied by species and strain, though were highest in either the lung or ileum in most cases.

**Table 2:** Number of DEGs in each treatment group (LogFC >1.5, FDR < 0.05).

	Brain			Ileum			Lung		
	Time 1	Time 2	Time 3	Time 1	Time 2	Time 3	Time 1	Time 2	Time 3
H9 infection									
Chicken	22	15	10	444	394	326	73	12	89
Turkey	451	726	349	53	687	1113	–	–	–
Duck	46	96	17	23	188	31	301	488	572
Crow	36	21	12	186	162	186	133	38	89
Goose	7	0	138	418	636	320	46	4	149
Pigeon	5	3	5	19	65	219	195	9	17
2.2 infection									
Chicken	73	223	892	305	275	744	464	1069	2124
Turkey	489	1135	675	697	223	304	–	–	–
Duck	24	95	498	24	141	268	534	790	444
Crow	48	54	16	652	341	228	75	273	113
Goose	294	23	260	331	43	279	71	57	20
Pigeon	6	4	21	10	55	93	94	37	348
2.3.2.1 infection									
Chicken	23	147	1342	238	1301	1930	266	596	1902
Turkey	36	372	1167	214	939	1500	–	–	–
Duck	46	360	607	10	275	406	100	822	402
Crow	21	19	38	54	117	116	249	319	204
Goose	86	237	245	121	326	161	17	68	32
Pigeon	13	12	5	23	26	33	45	67	34

Time 1, 2, 3 = 12h, 24h and 48h in chicken and turkey to 2.2 and 2.3 infection. Time 1, 2, 3 = 12h, 48h and 5d in the remaining treatment groups.

### 3.4 Species-specific responses

A profile of the response in each species was determined by analysis of DEGs (**Supplementary File 5**), GO terms associated with DEGs (**Supplementary File 6**) and expression of interferon stimulated genes (ISG) from RNA-seq data (**Figure 3**; **Supplementary File 7**). Additionally, qPCR was used to further examine and confirm innate immune gene expression in chicken (**Supplementary File 8**: **Figure S1**). The primary focus of this work was to examine the responses to different HPAI strains, so presented results will mainly compare the effects of 2.2 and 2.3 challenge.

#### 3.4.1 Chicken

Following HPAI 2.2 challenge, upregulation of immune pathways was not seen at 12h in any tissue (**Supplementary File 6**). In the lung and ileum at 12 and 24h, upregulated genes were primarily associated with hormone response, cell development and proliferation, and neuronal function pathways. At 24h only a limited immune response was seen in lung or brain, but there was significant upregulation of some ISGs and innate immune genes in the ileum such as *CATHB1*, *IL22* and *IFIT5*. This was confirmed by qPCR which showed clear upregulation of innate immune genes by 24h in ileum but not lung or brain. There was a very strong immune response in all three tissues by 48h, demonstrated by both RNA-seq and qPCR data. This involved a large upregulation of interferons (IFNs; up to 9-fold in the brain), ISGs, genes associated with both innate and adaptive immune response, apoptosis and viral defence pathways. HPAI 2.3 challenge induced a very similar response to that following 2.2, with a strong immune response at 48h in all tissues and with the same cytokines and pathways being upregulated. However, the immune activation occurred earlier than in response to 2.2 with ISGs upregulated in all three tissues by 24h, with particularly strong up-regulation in the ileum. This earlier immune response was seen both in RNA-seq and confirmed in the qPCR data.

#### 3.4.2 Turkey

Following HPAI 2.2 challenge an immune response was seen in the brain and ileum as early as 12h with strong up-regulation of several ISGs including *RSAD2* and *IRF7* (brain) and *MX1* (ileum). The immune response increased over time, with the highest up-regulation of immune pathways at 48h. In the ileum, pathways associated with metabolism and ion transport were differentially regulated across the course of infection. Following 2.3 challenge, as for chicken, the turkey response to 2.3 was very similar to that against 2.2, except that the immune response occurred earlier, with stronger up-regulation of ISGs at 24h in both brain and ileum in response to 2.3.

#### 3.4.3 Duck

HPAI 2.2 challenge induced an immune response in all tissues and included strong up-regulation of ISGs, defensins and cathelicidins. No immune pathways were significantly up-regulated at 12h though a small number of immune genes were, and immune response peaked in all tissues with the strongest up-regulation of gene expression at 48h. Many ISGs were still significantly up-regulated by 5d in all tissues (**Figure 3**; **Supplementary File 5**). This strong increase in expression of inflammatory genes across tissues including the brain was surprising, considering ducks show minimal clinical signs after challenge with 2.2. The host response to HPAI 2.3 challenge shared many of the same features as infection with 2.2, although the timing of immune response differed. Up-regulation of immune genes was not seen at 12h in the lung after 2.3 challenge as was seen with 2.2 challenge. However, as with 2.2, the ISG response generally peaked at 48h after challenge, but it was more sustained in the ileum, with ISGs still strongly up-regulated at 5d. There therefore may be a delayed immune response in the duck lung after challenge with clade 2.3.

#### 3.4.4 Crow

Following HPAI 2.2 challenge an immune response was already present in the ileum by 12h, mostly featuring many chemokines (*XLC1, CCL5, CXCL14*) and genes associated with T-cell or NK function (*CD3E/D, TNFSF8, EOMES*). The strongest ISG response in both the lung and ileum was at 48h. In the brain there was a modest up-regulation of ISGs, which disappeared by 5d. Following 2.3 challenge, ISGs were up-regulated in the lung at 48h, but not as strongly as with 2.2, and they remained up-regulated at 5d. In the ileum, immune response was first apparent at 48h and showed expression of a similar suite of genes as were activated by 2.2 at 12h. Very few genes were differentially regulated in the crow brain after 2.3 challenge. This included a few ISGs at 48h and 5d, but these were not as strongly expressed as when under challenge by the 2.2 virus. As with the duck, the crow response to 2.3 appears to be weaker and delayed, particularly in the lung and brain, compared to that of 2.2. This may help explain the poorer outcome following infection.

#### 3.4.5 Goose

Following HPAI 2.2 challenge there was little upregulation of immune genes seen in the lung, however at 12h and 48h there was a strong up-regulation of genes associated with nerve function (**Supplementary File 5**). In ileum most differentially expressed genes were related to ion transport and nerve function at 12h and 48h and to metabolic functions at 5d. In the brain there was little response until 5d when there was increased expression of a range of ISGs and genes associated with adaptive immunity. The immune response after challenge with 2.3 was stronger, with robust up-regulation of a range of ISGs at 48h in the brain and lung, with immune gene up-regulation maintained at 5d in the brain.

#### 3.4.6 Pigeon

Following HPAI 2.2 challenge, the strongest response was in the lung. There was up-regulation of genes associated with nerve function at 12h and 5d. In both lung and ileum there was down-regulation of some immune pathways at 5d (**Supplementary File 6**), including those of innate immunity and viral response. In the brain there was little response, however there were a few immune genes up-regulated including *AVD* and *CCL5*. The response to 2.3 was similar to that found for 2.2, with few DEGs and both up- and down-regulation of immune genes. Few genes were differentially regulated in lung, but one that was consistently strongly down-regulated was *OLFM4*, an anti-apoptotic gene. In the brain there were several complement-associated genes up-regulated at 12h and 48h. Notably, the baseline ISG expression in control pigeon samples appears substantially higher than that of other birds, in both lung and ileum.

### 3.5 Cross-species comparison

**Figure 4:**
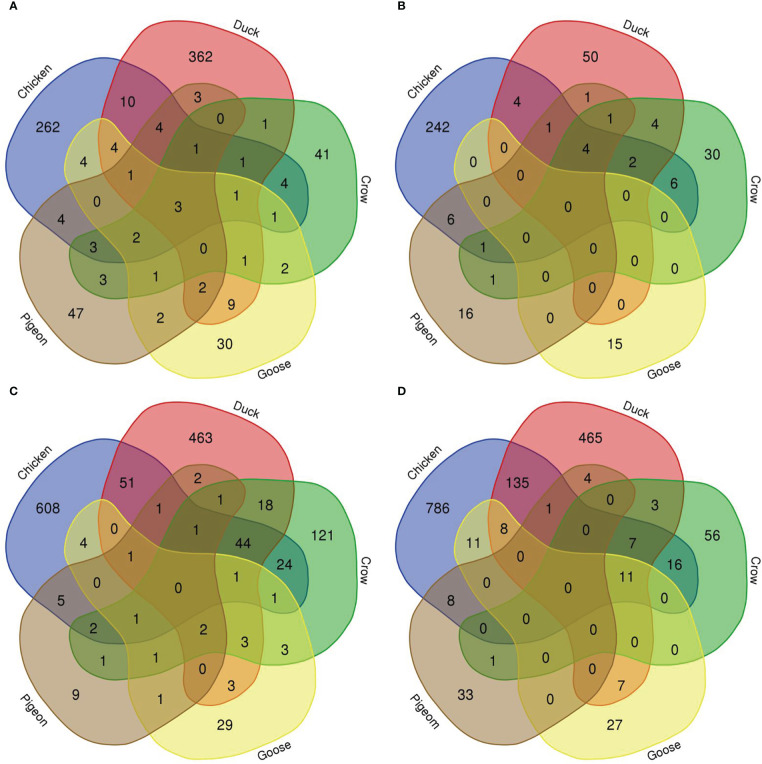
Venn diagram of genes differentially expressed in lungs in each species. **(A)** 12h 2.2, **(B)** 12h, 2.3, **(C)** 48h 2.2, **(D)** 48h 2.3.

To compare the response to H5N1 infection across the six species we firstly constructed Venn diagrams to identify genes uniquely or commonly differentially regulated in each of the six bird species (**Figure 4**; **Supplementary File 8**; **Figures S2, S3**). Across the treatment groups the majority of differentially expressed genes were uniquely differentially expressed in only one species. There were also many common DEGs between two or more of the four most susceptible bird species (chicken, turkey, duck and crow), and these were most frequently immune genes and ISGs, likely a response to the higher viral loads seen in these species. There were few common DEGs shared between goose, pigeon and the remaining four species, although goose DEGs were more commonly shared with duck than the remaining species, likely due to their close evolutionary relationship.

We further examined DEGs from groupings of interest, including those with a possible role in resistance/susceptibility. Of particular interest were any DEGs in all species, DEGs from only the resistant species, and DEGs unique to pigeon and goose (the most resistant species). These genes are shown in **Table 3**. The majority of these genes were associated with two main functions and are thus highlighted in the table - immune genes (blue) and those associated with the nervous system (orange). Most genes in these groups were immune genes, largely ISGs and anti-viral genes, and these were often common to all species, or all those excluding pigeon. Other genes of particular interest that were differentially expressed in multiple species included *OLFM4*, an anti-apoptotic gene which is also involved in innate immune response regulation (Zhang et al., 2004) that was often differentially expressed exclusively in the more resistant species. *GUCA2B* is a gene with a role in water and salt transport in intestine and was expressed more highly in resistant species. *BPI*, an antimicrobial gene with a known role in innate immunity and which can inhibit the infectivity of influenza (Pinkenburg et al., 2016), was also found to be differentially expressed only in pigeon and goose.

**Table 3:** Common DEGs in key comparisons.

Chicken, Crow, Duck, Goose, Turkey	CMPK2,EPSTI1,IFIT5,IFITM1,MX1,OASL,RSAD2,SIGLEC1,USP18,ZNFX1
Chicken, Crow, Duck, Goose, Pigeon	GRIN1,SNAP25,PLP1
Chicken, Crow, Duck, Turkey	C1QA,C1S,CHRDL2,CMPK2,DHX58,EPSTI1,IFI6,IFIT5,IGF2BP3,IL4I1,IRF1,IRF7,PARP14,LOC422513,LOC770612,MOV10,MX1,PARP9,RNF213,RSAD2,S100A9,SAMD9L,STAT1,TRIM25,USP18,ZC3HAV1,ZNFX1
Chicken, Duck, Goose, Turkey	C1QB,C3H8ORF80,CASP7,EIF2AK2,HELZ2,IFI35,IFIH1,IRF1,IRF7,PMLL,IFITM2,LOC422513,MITD1,MOV10,NLRC5,NMI,PARP12,PARP14,PARP9,PLACL2,RBM43,RNF213,SAMD9L,SOCS1,STAT1,TAP1,TAP2,TLR3,TMEM140,TRIM25
Chicken, Crow, Duck, Goose	LOC422513,CMPK2,SAMD9L,HELZ2,USP18,EPSTI1,OASL,RNF213,IFIT5,RSAD2,ZNFX1,CCL19,SLC17A6
Chicken, Crow, Duck, Pigeon	GUCA2B,MEP1A,FCGBP,FABP6,AGT
Chicken, Duck, Goose, Pigeon	HMP19
Chicken, Crow, Goose, Pigeon	CAMK2A,GPM6A
Crow, Duck, Goose, Pigeon	GUCA2B,OLFM4
Crow, Duck, Goose	ALDOB,SNAP25,SLC17A6,PLP1
Duck, Goose, Pigeon	GUCA2B,OLFM4,SLC17A6,GRIN1
Crow, Goose, Pigeon	GAP43,GPM6A,PVALB
Crow, Duck, Pigeon	AGT,OLFM4
Goose, Pigeon	GPM6A,SLC4A10,SLC17A6,GRM3,BPI,SCN2A

Common regulation identified in each individual treatment group and then pooled. Immune genes are colored blue and genes associated with nerve-related function are shown in orange.

**Figure 5:**
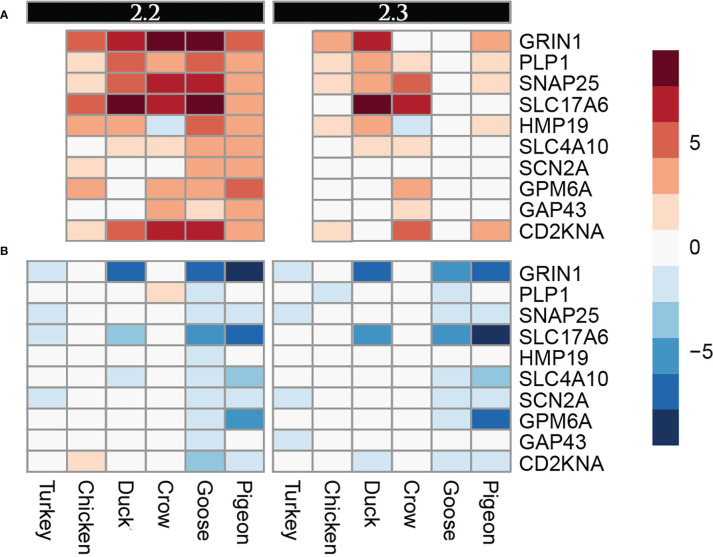
Heat map diagram of differential expression of selected nervous function genes at 12h in **(A)**. Lung and **(B)**. Ileum. Legend is logFC relative to control samples.

A group of genes associated with nerve cells and their function were often commonly differentially regulated in multiple species, but particularly in goose and pigeon. Surprisingly this set of genes was not differentially regulated in brain (except for *PLP1*, down-regulated in duck at 5d), instead showed strong differential regulation in both lung and ileum. This group of genes encode proteins including GRIN1, which plays a key role in the plasticity of synapses, PLP1 which is the predominant component of myelin and SNAP25 which is involved in the regulation of neurotransmitter release. To look at this group further a heat map of these identified nerve-associated genes at 12h (**Figure 5**) and 48h was constructed (**Supplementary File 8**; **Figure S4**). A very similar expression pattern was seen at both time points. In the lungs, these nerve-associated genes are frequently up-regulated following infection. After challenge with 2.2, the up-regulation of these genes was much higher in the resistant species, with some genes expressed up to 9-fold higher, some of the most highly up-regulated genes following infection (**Supplementary File 5**). After 2.3 challenge, these showed the highest up-regulation in crow and duck. In the ileum these genes were conversely down-regulated following infection, and again the effect was stronger in the resistant birds, with genes showing up to 10-fold down-regulation. In the ileum, the highly resistant goose and pigeon showed the strongest down-regulation of these nervous system genes, in both 2.2 and 2.3 infections. These data show a complex interaction between tissue, species and strain of virus, but with the strongest differential regulation in the more resistant species.

**Figure 6:**
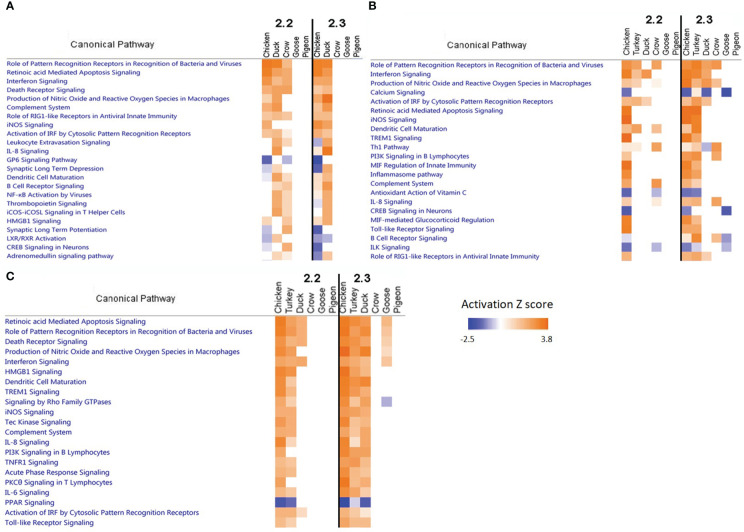
Pathway analysis comparison in **(A)** Lung, **(B)** Ileum and **(C)** Brain.

To further examine patterns of gene pathway and network regulation we examined the data from all species in a pathway analysis using IPA software. We compared the regulation of pathways in each tissue (**Figure 6**; **Supplementary File 8**; **Figure S5**), focussing on the 48h time point as this showed the strongest pathway enrichment. In the lung at 48h we can see that strongest enrichment of pathways during 2.2 infection was in chicken, duck and crow, consistent with the greater number of DEGs seen in these species. These were largely immune-related pathways such as pattern recognition, interferon signalling, complement system and RIG-I receptor signalling pathways. Crow did not show the same strong enrichment of immune pathways at 48h after 2.3 infection, consistent with their weaker but more extended up-regulation of immune genes, as discussed earlier.

In the ileum a similar pattern was seen as in lung, with strongest enrichment of pathways in the chicken, turkey, duck and crow with mostly the same immune-related pathways being affected. Down-regulation of calcium signalling and CREB signalling in neuronal pathways were related to the down-regulation of nerve-associated genes.

In the brain the strongest enrichment of pathways was in the chicken and turkey in response to 2.2 challenge, and additionally duck to 2.3 challenge. This included a host of immune and inflammatory pathways, including apoptosis signaling, pattern recognition, interferon signaling and death receptor signaling. This strong inflammatory response is likely a reaction to the high viral load in the brain of these species, but may also be contributing or causing the neurological damage seen in these species. The duck in particular showed a much stronger enrichment of inflammatory pathways in response to 2.3 compared to 2.2, which may underlie the differing susceptibility of ducks to these two infections. Lastly, the goose also showed some up-regulation of these inflammatory pathways in the brain, despite the resistance of this species and the relatively low viral load seen in the brain. This indicates that the 2.3 virus may have a greater impact than 2.2 on the brain and nervous system of birds, even when hosts are relatively resistant and show no symptoms.

### 3.6 Comparison of 2.2 vs 2.3 challenge in duck and crow

**Figure 7:**
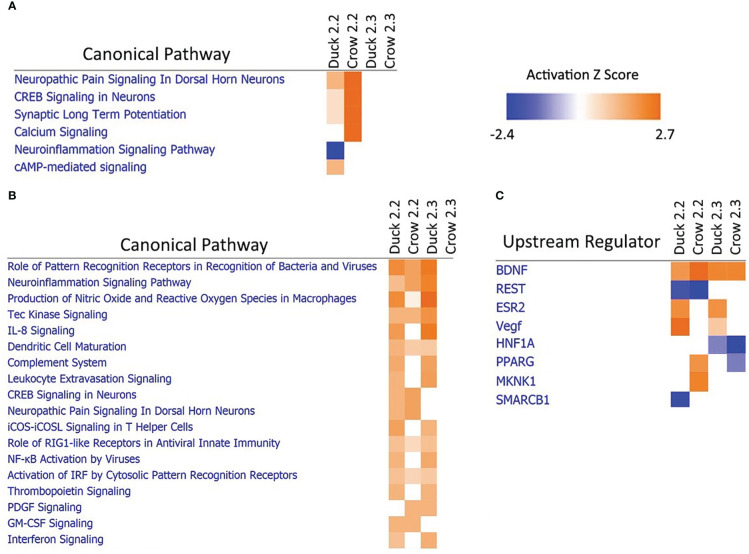
Pathway analysis of crow and duck lung at **(A)** 12h and **(B)** 48h and upstream regulators at 12h **(C)**.

Due to the difference in outcomes after 2.2 and 2.3 infections in both duck and crow (Khan et al., 2014; **Supplementary File 3**), we compared the host responses in more detail using a pathway approach. A pathway comparison (**Figures 7A, B**; **Supplementary File 8**; **Figure S6, S7**) showed the clearest distinction between 2.2 and 2.3 response in the lung. At 48h, immune pathways were strongly enriched after challenge by both strains in duck but only against 2.2 in crow. As discussed previously, the response to 2.3 was lower and delayed in crow. At 12h in these species however, it was neuronal-related pathways that were strongly enriched. Intriguingly, this was seen in response to 2.2 but not 2.3 in both species. To understand this further we looked at the upstream regulators predicted to control these pathways (**Figure 7C**). Two regulators stood out as showing a strong difference between the 2.2 and 2.3 infections. Firstly, the regulatory gene *BDNF* was enriched in both species in response to each strain. This gene supports survival of neurons and encourages growth and differentiation of new neurons and synapses (Duman, 1999). A second gene regulator, *REST*, was suppressed specifically in response to 2.2 but not 2.3 virus. REST is a transcriptional repressor that suppresses neuronal gene expression in non-neuronal tissues (Chong et al., 1995). Suppression of this regulator can therefore be predicted to increase expression of neuronal genes in tissues such as the lung. The interaction of these regulators may be responsible for the differential regulation of neuronal pathway genes following HPAI infection, and furthermore may contribute to the differing outcome of duck and crow to 2.2 and 2.3 infection.

### 3.7 Host response genes shared between birds and humans

To further examine similarities in response between the six species we analysed the data using a meta-analysis by information content (MAIC) approach (Li et al., 2020). This analyses the shared information content across species and produces a ranked list of genes summarising the combined evidence from species of each gene being involved in HPAI response (**Supplementary File 8**, **Figure S8**). This demonstrated that the top genes shared across these species were largely ISG genes, consistent with other analyses which found strong ISG responses in these species. We also extended this analysis by including data from human influenza studies, including data from CRISPR/CAS9 screen, annotated pathways, genetic perturbation screens and protein–protein interactions (See Li et al., 2020 for full list of data sources included; **Supplementary File 8**, **Figure S8**). This study found strong shared information content between the data from this current study and that of many human studies. The top ranked genes were primarily ribosomal genes and others (including *MCM7* and *FASN*) that have been reported to act as important host factors aiding virus genome replication in host cells (Kawaguchi and Nagata, 2007; Du et al., 2020).

### 3.8 Candidate gene identification

Lastly, it was a goal of this study to identify candidates which can be used in future investigation using *in vitro* systems and animal modification experiments to elucidate their role in HPAI susceptibility. From this study we have identified a list of genes that may have a role in resistance (**Table 4**). These were identified either due to a differential response in resistant and susceptible birds, a differential response to 2.2 vs 2.3 in ducks and crows, or a unique response in pigeon, being the species exhibiting the highest resistance. We have included several of the most strongly differentially expressed nerve-associated genes and regulators, including *GRIN1*, *SNAP25*, *SLC17A6* and *REST. OLFM4* is another key candidate discussed previously; this gene has anti-apoptotic function, is a stem cell marker associated with cell proliferation, and is involved in the regulation of host innate immunity (Zhang et al., 2004). Furthermore high expression of this gene is associated with poor prognosis in several diseases in humans (Liu and Rodgers, 2022). *OLFM4* showed strong downregulation in resistant species in the face of challenge with both 2.2 and 2.3 viruses. We identified several antimicrobial peptides, both defensins (*AvDB2*, *AvBD10*) and cathelicidins (*CATH2*) that showed different responses in duck and crow to 2.2 vs 2.3 infection. We also identified a range of genes, including *PERP1* a pro-apoptotic cytokine and *A4GNT* which is involved in mucin synthesis, which were exclusively differentially regulated in the highly resistant pigeon or goose. These could be interesting targets for further research.

**Table 4:** Candidate genes with possible roles in resistance/susceptibility.

Gene Symbol	Function	Tissue	Differential response
OLFM4	Anti-apoptotic factor. Regulates host innate immunity. Extracellular matrix glycoprotein that facilitates cell adhesion	Lung	Stronger down-regulation in resistant species for both 2.2 and 2.3
GRIN1	Part of ligand-gated ion channel, plays a key role in the plasticity of synapses	Lung, Ileum	Stronger differential regulation in resistant species for both 2.2 and 2.3
SNAP25	Presynaptic plasma membrane protein involved in the regulation of neurotransmitter release	Lung, Ileum	Stronger differential regulation in resistant species for both 2.2 and 2.3
SLC17A6	Mediates the uptake of glutamate into synaptic vesicles at presynaptic nerve terminals of excitatory neural cells	Lung, Ileum	Stronger differential regulation in resistant species for both 2.2 and 2.3
REST	Transcriptional repressor that represses neuronal genes in non-neuronal tissues	Lung, Ileum	Possible regulator of nerve-function genes showing differences between resistant and susceptible species
AvBD2	Defensin, antimicrobial	Lung	Up-regulated after 2.2 infection, not 2.3 in Duck and Crow
AvBD10	Defensin, antimicrobial	Lung	Up-regulated after 2.2 infection, not 2.3 in Duck and Crow
GSDMA	Triggers cell death. Also binds to bacterial and mitochondrial lipids	Brain	Up-regulated after 2.2 infection, not 2.3 in Duck and Crow
CERKL	Overexpression protects cells from apoptosis in oxidative stress conditions	Lung	Up-regulated after 2.2 infection, not 2.3 in Duck and Crow
TRIM29	May act as a transcriptional regulatory factor	Ileum	Down-regulated after 2.3 infection, not 2.2 in Duck and Crow
A4GNT	GlcNac transferase, Necessary for the synthesis of type III mucin	Lung	Up-regulated in pigeons, not in any other species
HAAO	Role in viral mRNA translation	Lung	Down-regulated in pigeons, not in any other species
PERP1	Regulation of antibody secretion, diversifies B-cell function and proapoptotic	Lung	Down-regulated in pigeons, not in any other species
TRPV4	Regulates expression of chemokines and cytokines related to proinflammatory pathway	Ileum	Up-regulated in pigeons, not in any other species
CATH2	Antimicrobial, binds LPS, might have a role in innate responses	Lung	Up-regulated after 2.2, not 2.3, in ducks
CXCR1	Receptor for IL-8, activates neutrophils/heterophils	Lung	Up-regulated after 2.2, not 2.3, in ducks
SLA2	Negative regulator of T-cell signalling	Lung	Up-regulated after 2.2, not 2.3, in ducks

## 4 Discussion

Understanding the underlying molecular and cellular response to avian influenza infection in resistant and susceptible avian species is crucial for mitigating the impact of this economically devastating disease. In this study we have analysed the gene expression profiles of six avian species with varying susceptibility to two strains of HPAI and identified key patterns, similarities and differences in the responses of these host species.

The first major difference between the species tested in this study was the disease progression and viral loads. Our pilot data confirmed the high tolerance of the geese and pigeons to HPAI infection, showing no symptoms or mortality in the test period. We also demonstrated that while all six species showed evidence of viral replication, the viral loads were much higher in susceptible species, with high viral loads in the lung, ileum and brain as early as 12 hours in susceptible species.

Our study is the first to compare the host responses in the brain in these six species. Chickens show neurological symptoms following infection with HPAI, and it has been proposed that infection of the central nervous system may contribute to the high mortality in poultry species and wild birds (Sturm-Ramirez et al., 2004; Tanimura et al., 2006; Zou et al., 2010; Balasubramaniam et al., 2012). We found very high viral loads in the brains of chickens and turkeys in this study, and in ducks after 2.3 infection. This was accompanied by strong upregulation of ISGs, pro-inflammatory cytokines and other immune genes, along with enrichment of inflammatory pathways. Prior studies have found inflammation, apoptosis, gliosis and neuronal degeneration in the brain of chickens, ducks and crows following HPAI H5N1 infection. (Fleming-Canepa et al., 2019; Kumar et al., 2020). By contrast, the viral loads in the brain of crow, goose and pigeon were low, and they showed little to no up-regulation of inflammatory cytokines. The high viral loads and corresponding neuro-inflammation in susceptible species is likely to be the cause of neurological symptoms and damage to the brain, resulting in high morbidity and mortality seen in these species.

The duck and crow were key species in this study, as they typically show high tolerance to HPAI infections, but have high mortality when specifically faced with the 2.3 viral strain (Khan et al., 2014). Some key differences were identified that may explain this increased mortality. Firstly, both species showed a delayed immune response to 2.3. Both species showed immune gene up-regulation by 12 hours in response to 2.2 challenge: duck in the lung and crow in the ileum. However, immune gene up-regulation was not seen at 12 hours in either species after 2.3 challenge. This lack of response at 12 hours cannot be explained by viral load, with both crow and duck showing no significant difference in viral load between 2.3 and 2.2 at this time point, in all tissues. The up-regulation of ISGs was more sustained in response to 2.3 in both species, in both lung and ileum, with stronger immune gene up-regulation at 5 days, likely an indication that the birds have not overcome and recovered from the infection by 5 days. Prior studies have also found delayed timing of immune responses in species with higher susceptibility (Cornelissen et al., 2013), but this is the first time delayed timing has been shown with different strains of HPAI in the same species. This delayed immune response likely contributes to the higher mortality seen in ducks and crows in the face of 2.3 challenge. Further research into the early interaction between virus and these species would be useful for exploring the cause of this delayed response.

The goose and pigeon are two species observed to be highly resistant to H5N1 infection (Yamamoto et al., 2012) and this was confirmed by our animal trials. As well as no observed mortality from both the 2.2 and 2.3 strains, these birds also showed very low viral loads following infection, in particular the pigeon. Both species showed low up-regulation of immune responses following infection, again particularly the pigeon. This suggests that the virus may be blocked by innate factors or barriers that prevent viral entry into cells or spread of the virus. One interesting finding was the higher overall ISG expression in the pigeon control samples compared to other species. It is possible that this higher baseline expression of many ISGs could be providing a protective effect. Alternatively, as these birds were acquired from markets, the possibility of pre-existing infection or diseases cannot be entirely ruled out, despite best efforts to check for disease and parasites in the acquired birds. In the goose stronger immune response was seen following 2.3 challenge compared to 2.2 challenge, and even including inflammatory response in the brain, indicating that even in species highly tolerant of both strains, 2.3 does elicit a stronger immune response.

One unexpected finding of our analysis was the strong differential regulation of many genes associated with nerve function in both the lung and ileum. The observed expression pattern was that of up-regulation in the lung and down-regulation in the ileum, with much stronger differential regulation in resistant species. Furthermore, this up-regulation was weaker in ducks and crows after 2.3 challenge compared to that of 2.2, suggesting an intriguing viral strain interaction. These genes were some of the strongest differentially regulated genes in this study, with up to 10-fold up-regulation or down-regulation, occurring as early as 12 hours post infection, suggesting a very strong and rapid reaction in these pathways. Ours is not the first study to find a link between nerve-associated genes and avian influenza. Zou et al. (2010) found significant changes in gene expression in neural signal transduction proteins, while a GWAS study (Drobik-Czwarno et al., 2018) identified three nerve-related genes which were candidates for differing susceptibility to HPAI in chickens. As previously discussed, the rapid influx of HPAI virus in the central nervous system (CNS) may be critical for the high susceptibility and mortality of chickens to HPAI. Studies in ferrets and mice have demonstrated influenza may enter the CNS through the olfactory, vagal, trigeminal, and sympathetic nerves (Park et al., 2002; Plourde et al., 2012; Shinya et al., 2000). Regulation of peripheral nerve function in tissues may be one means of controlling viral spread to the central nervous system. Additionally, there is a growing body of research on the effect of the neuro-immune interaction, particularly in the gut (Keita and Söderholm, 2010). In intestinal mucosa there is close connections between nerve cells and immune cells, with immune cells such as mast cells responding to neural stimulation (Stead, 1992). This cross-talk between the nervous and immune system has largely been studied in the context of stress, nematode parasitism, bacterial infections, respiratory disease and food sensitivity (Pothoulakis et al., 1998; Keita and Söderholm, 2010; Chu et al., 2020; Camp et al., 2021), but perhaps this neuro-immune interaction also plays a key role in infectious viral disease. Investigating the role of peripheral nerves in mucosal tissues in influenza infections could be an intriguing target for future research.

In this study we identify several candidate genes with possible roles in resistance/susceptibility. As previously mentioned, a suite of nerve-associated genes showed stronger differential regulation in the more resistant species and showed a difference in expression against the two different HPAI viral strains. We identified the genes *REST* and *BDNF* as two possible regulators of these nerve genes; the latter gene supports survival of neurons and encourages growth and differentiation of new neurons and synapses (Duman, 1999), and was enriched in resistant species in response to both strains of virus. REST, a transcriptional repressor that limits neuronal gene expression in non-neuronal tissues (Chong et al., 1995), was suppressed during 2.2 infection but not 2.3. Another interesting gene that was identified through multiple analyses was *OLFM4*, an anti-apoptotic factor that is also involved in cell adhesion and proliferation (Zhang et al., 2004). This gene was down-regulated in the lung of all species, but was more strongly down-regulated in resistant species. As apoptosis has previously been identified as a key difference between the response of ducks and chickens to H5N1 (Cornelissen et al., 2013) and between the response to LPAI and H5N1 (Hui et al., 2016), this gene may be a key controller of this resistance mechanism. Several other genes were identified that were up-regulated only in response to 2.2 infection and not 2.3 infection in ducks and crows. This included several defensins - peptides which can have direct anti-viral activity and can also play a role as important immune signalling molecules (Ding et al., 2009), and thus could play a key role early in infection. We also identified genes that were uniquely differentially expressed in the pigeon, the most resistant of the six birds in this study. These genes had a variety of functions that may be involved in inhibition of viral replication and spread, including *A4GNT* a gene involved in the synthesis of type II mucin (Nakayama et al., 1999) and *PERP* which is involved in the regulation of apoptosis (Attardi et al., 2000). Investigating the role of these genes in HPAI infection in more depth would be an excellent target for future research.

## 5 Conclusions

In this study, we investigated the response of six bird species with varying susceptibility to two strains of HPAI H5N1. We discovered intriguing patterns in the differential responses of these species which helps to elucidate the differing mortality and morbidity rates in these species. We found high viral loads and strong up-regulation of neuro-inflammatory pathways in the brains of susceptible species, explaining the neurological symptoms seen in these species. Linked to this, we found a surprising strong differential regulation of genes associated with nerve function in the lung and ileum. Regulation of peripheral nerves at the mucosal sites could potentially be linked to the transmission of the virus to the brain through peripheral nerves or be related to neuro-immune regulation in the mucosa to control viral infection and spread. Additionally, we found key differences in the timing of immune response to different strains of HPAI in ducks and crows, which may explain the differing mortality rates seen in response to these two strains in these species. Lastly, we identified a panel of candidate genes which can be used for future research to further explore their role in HPAI susceptibility and could be targets for selective breeding or gene editing in the development of domestic birds with increased HPAI resistance. The knowledge gained from this study can be used to develop sustainable strategies to control HPAI infections in domestic poultry in the future.

## Data availability statement

The datasets generated for this study can be found in the Short Read Archive at the National Center for Biotechnology Information (NCBI) [https://www.ncbi.nlm.nih.gov/sra] under accession number PRJEB41063.

## Ethics statement

All animal experiments were approved by the ICAR institute animal ethics committee (IAEC) approval (73/IAEC/HSADL/13).

## Author contributions

DWB, PD, LV, AM and AR conceived and designed the study; AM, AR, PV, SC and RD conducted pilot and infection trials and collected the samples; EG performed qPCR analysis on viral genes; DB performed qPCR analysis on host genes; RK conducted Kallisto analysis on the RNA-seq data; BW and KB performed MAIC analysis; KM performed differential expression analysis and all other downstream analysis of the data, prepared the figures and wrote the manuscript; JS and DWB supervised the project and revised the manuscript. All authors contributed to the article and approved the submitted version.

## Supplementary material

Supplementary File 1 Number of samples in each treatment group in RNA-seq data.

Supplementary File 2 List of primers used for qPCR experiments.

Supplementary File 3 Result of pilot infection trials in crow, geese and pigeons.

Supplementary File 4 Result of TukeyHSD test on viral loads.

Supplementary File 5 DEGs in each treatment group.

Supplementary File 6 Results of PANTHER over-representation test.

Supplementary File 7 List of ISGs.
