# Corrigendum: The molecular basis of differential host responses to avian influenza viruses in avian species with differing susceptibility

**DOI:** 10.3389/fcimb.2023.1194878

**Published:** 2023-04-04

**Authors:** Katrina M. Morris, Anamika Mishra, Ashwin A. Raut, Eleanor R. Gaunt, Dominika Borowska, Richard I. Kuo, Bo Wang, Periyasamy Vijayakumar, Santhalembi Chingtham, Rupam Dutta, Kenneth Baillie, Paul Digard, Lonneke Vervelde, David W. Burt, Jacqueline Smith

**Affiliations:** ^1^ The Roslin Institute and R(D)SVS, The University of Edinburgh, Edinburgh, United Kingdom; ^2^ National Institute of High Security Animal Diseases, Indian Council of Agricultural Research, Bhopal, India

**Keywords:** avian influenza, transcriptome, H5N1, chicken, duck, pigeon, crow, disease resistance

## Error in author list

In the published article, there was an error in the named corresponding authors, and author Anamika Mishra was erroneously excluded. The corrected corresponding author list appears below.

Katrina M. Morris* - katrina.morris@roslin.ed.ac.uk; katrina.m.morris345@gmail.com

Anamika Mishra* - reach2anamika@yahoo.com

The authors apologize for this error and state that this does not change the scientific conclusions of the article in any way. The original article has been updated.

