# Supplementary material for: The molecular basis of differential host responses to avian influenza viruses in avian species with differing susceptibility
Source: Front Cell Infect Microbiol. 2023 Feb 28;13:1067993. doi: 10.3389/fcimb.2023.1067993 (PMC10011077; doi:10.3389/fcimb.2023.1067993)
Supplement: Supplementary File 1 — Number of samples in each treatment group in RNA-seq data. [file DataSheet_1.zip › Supplementary files/Supplementary File 3.docx]

**Results of virus shedding and seroconversion. 4 birds were infected and observed in each case**

| **Species** | **Virus** | **Oral shedding** | **Cloacal Shedding** | **Seroconversion**  **At 10dpi** |
| --- | --- | --- | --- | --- |
| **Crow** | H5N1 Clade 2.2 | 2dpi onwards in all  4/4 birds | 2dpi onwards in all  2/4 birds | nil |
|  | H5N1 Clade 2.3.2.1 | 3dpi onwards  4/4 birds | 3dpi onwards  3/4 birds | 1/3 at 12 dpi  HI titre 2^9^ |
| **Geese** | H5N1 Clade 2.2 | 2dpi onwards in all  2/4 birds | 2dpi onwards in all  1/4 birds | nil |
|  | H5N1 Clade 2.3.2.1 | 2dpi onwards in all  3/4 birds | No cloacal shedding  Up to 9dpi | 4/4 at 10 dpi  HI titre 2^6^ to 2^9^ |
| **Pigeon** | H5N1 Clade 2.2 | No shedding up to 9dpi | No shedding  Up to 9dpi | 3/4 with  HI titre 2^5^ |
|  | H5N1 Clade 2.3.2.1 | No shedding up to 9dpi | 2dpi onwards  1/4 birds | No seroconversion |
