# Supplementary material for: The molecular basis of differential host responses to avian influenza viruses in avian species with differing susceptibility
Source: Front Cell Infect Microbiol. 2023 Feb 28;13:1067993. doi: 10.3389/fcimb.2023.1067993 (PMC10011077; doi:10.3389/fcimb.2023.1067993)
Supplement: Supplementary File 1 — Number of samples in each treatment group in RNA-seq data. [file DataSheet_1.zip › Supplementary files/Supplementary File 8.docx]

**Supplementary Figures**


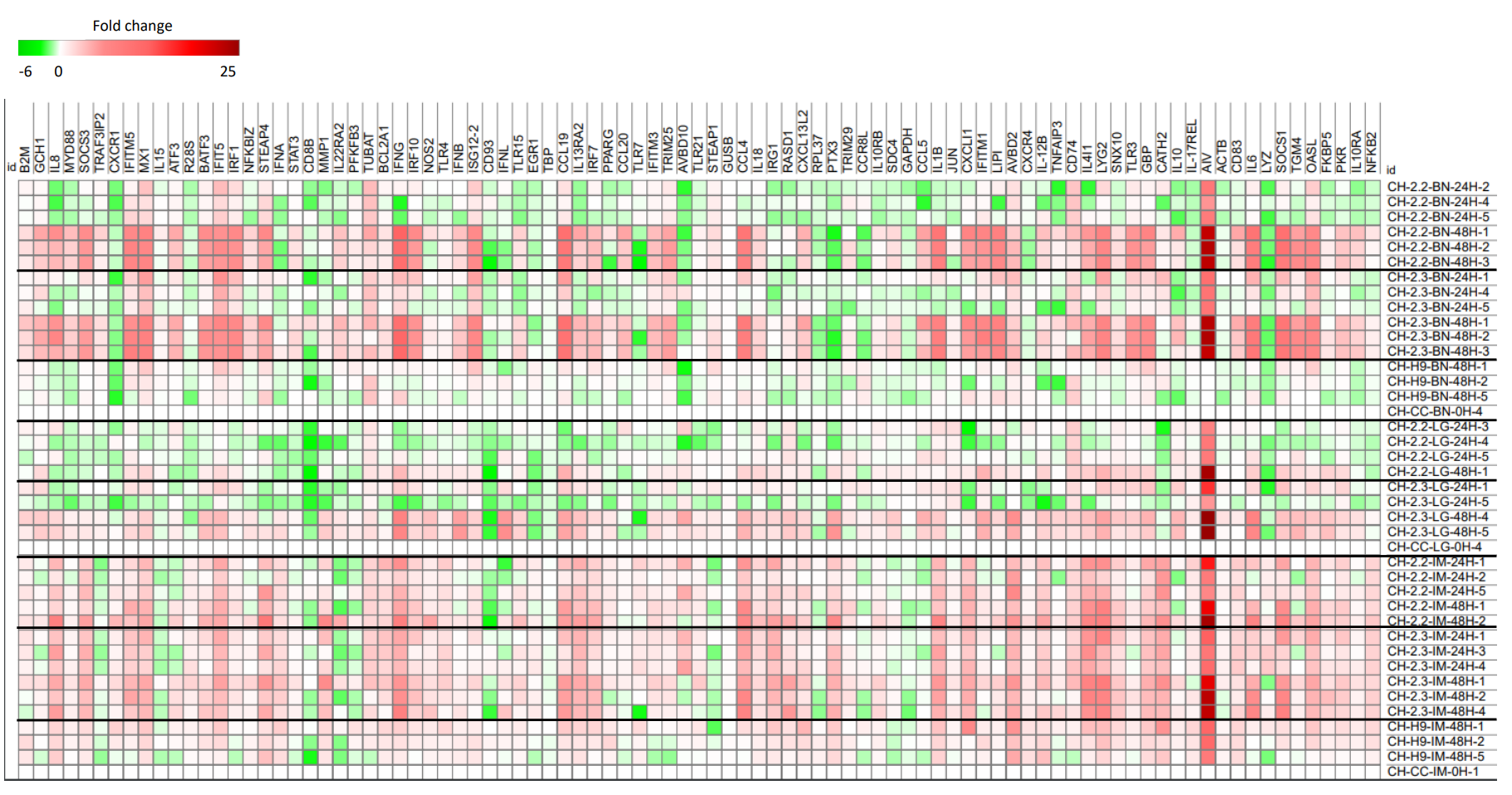


**Figure S1:** Heat map representation of changes in gene expression in brain, lung and ileum from AIV infected chickens from qPCR analysis. The expression of AIV gene is included here.


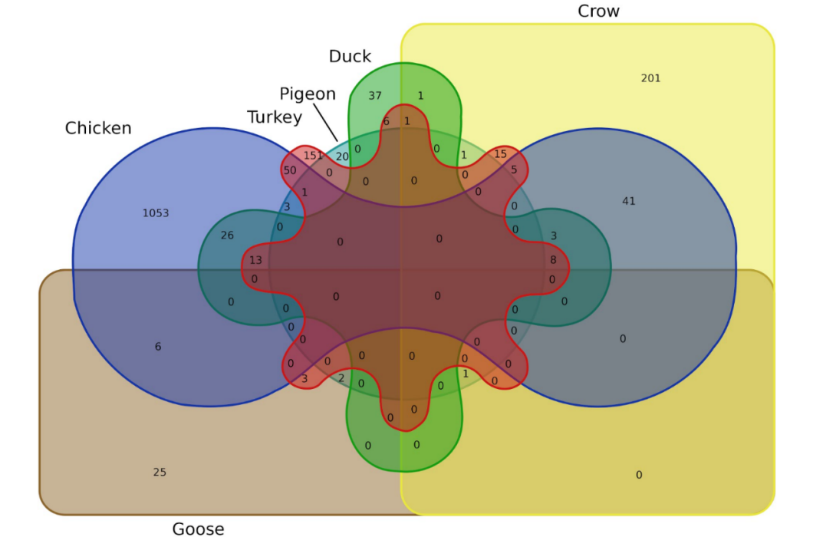

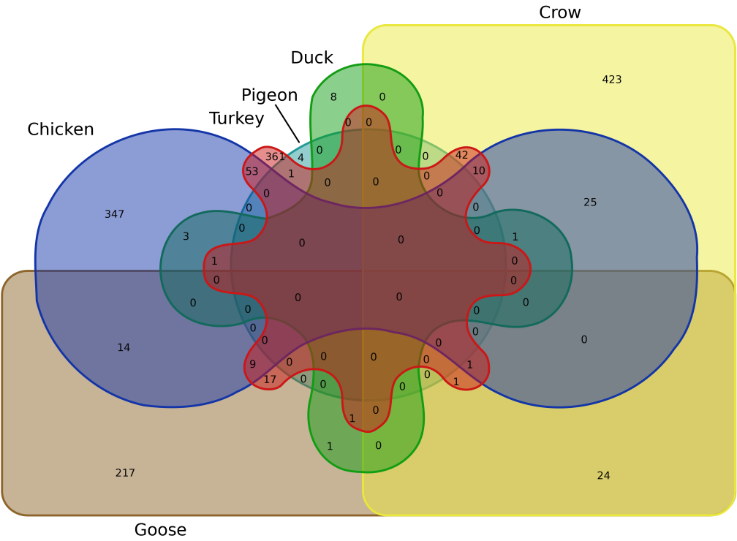

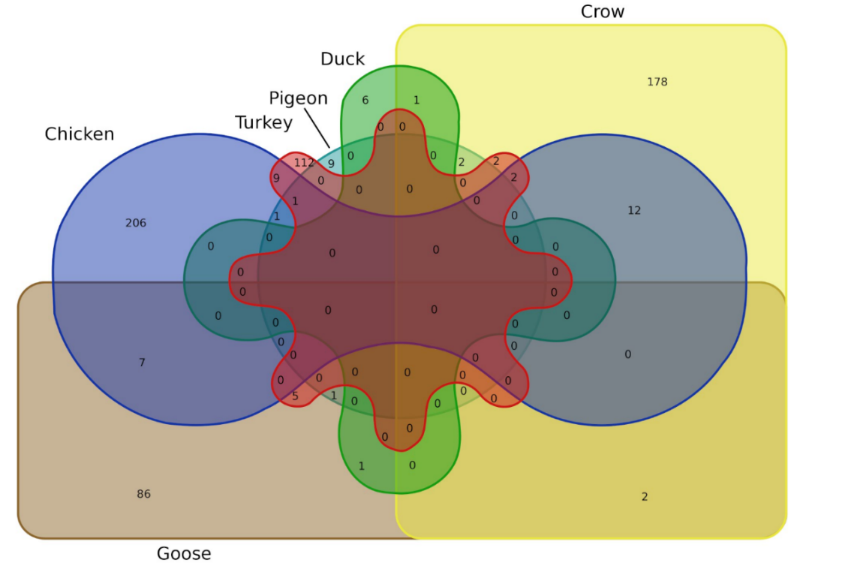

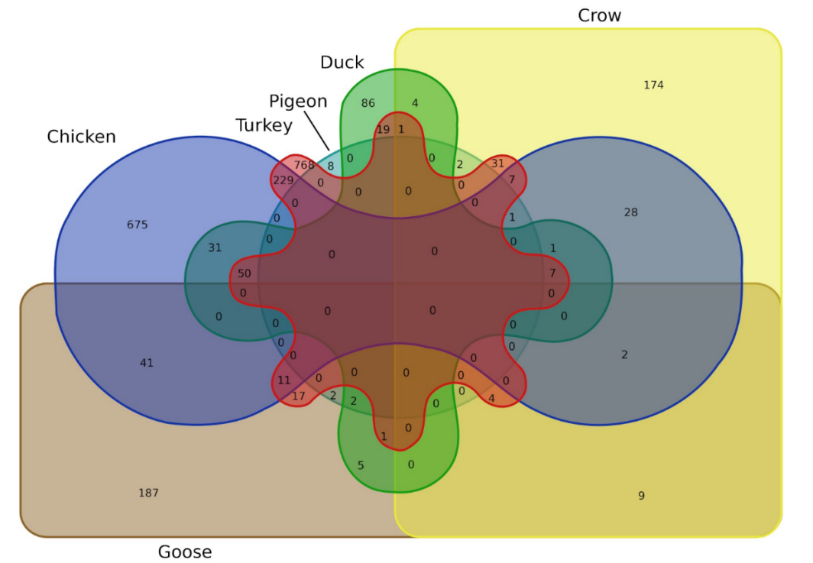


**D**

**C**

**B**

**A**

**Figure S2:** Venn diagram of genes differentially expressed in ileum in each species. (A) 12H 2.2, (B) 12H, 2.3, (C) 48H 2.2, (D) 48H 2.3.


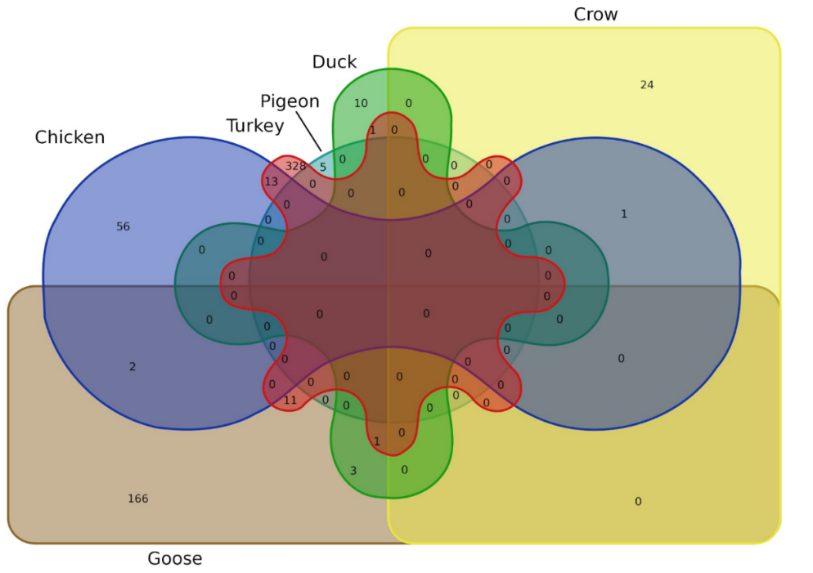

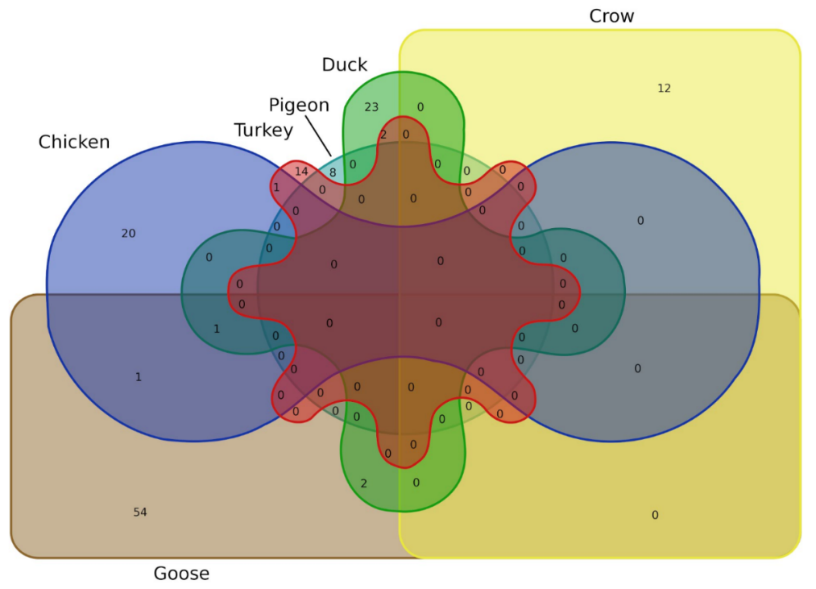

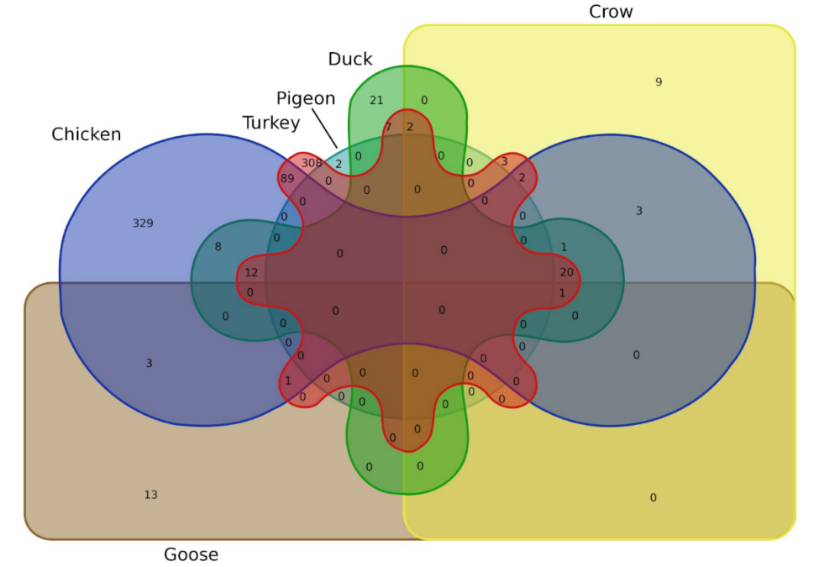

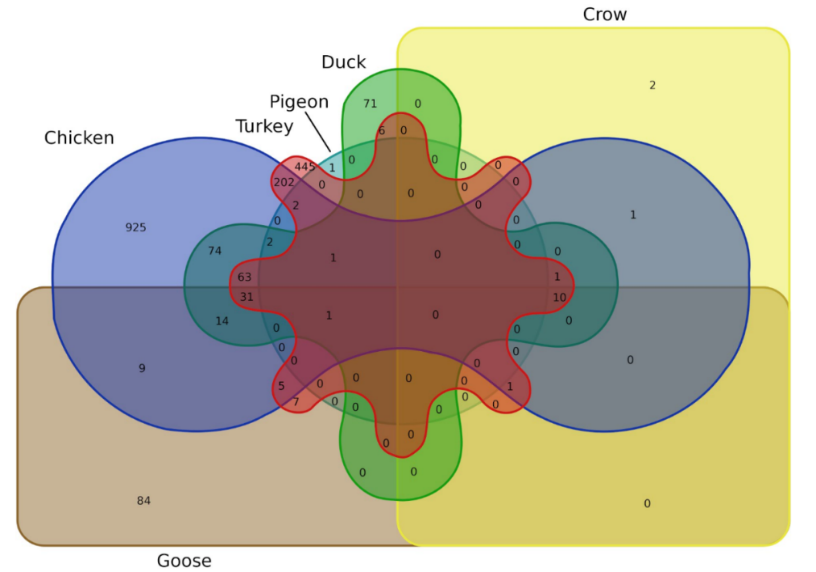


**D**

**C**

**B**

**A**

**Figures S3:** Venn diagram of genes differentially expressed in brain in each species. (A) 12H 2.2, (B) 12H, 2.3, (C) 48H 2.2, (D) 48H 2.3.


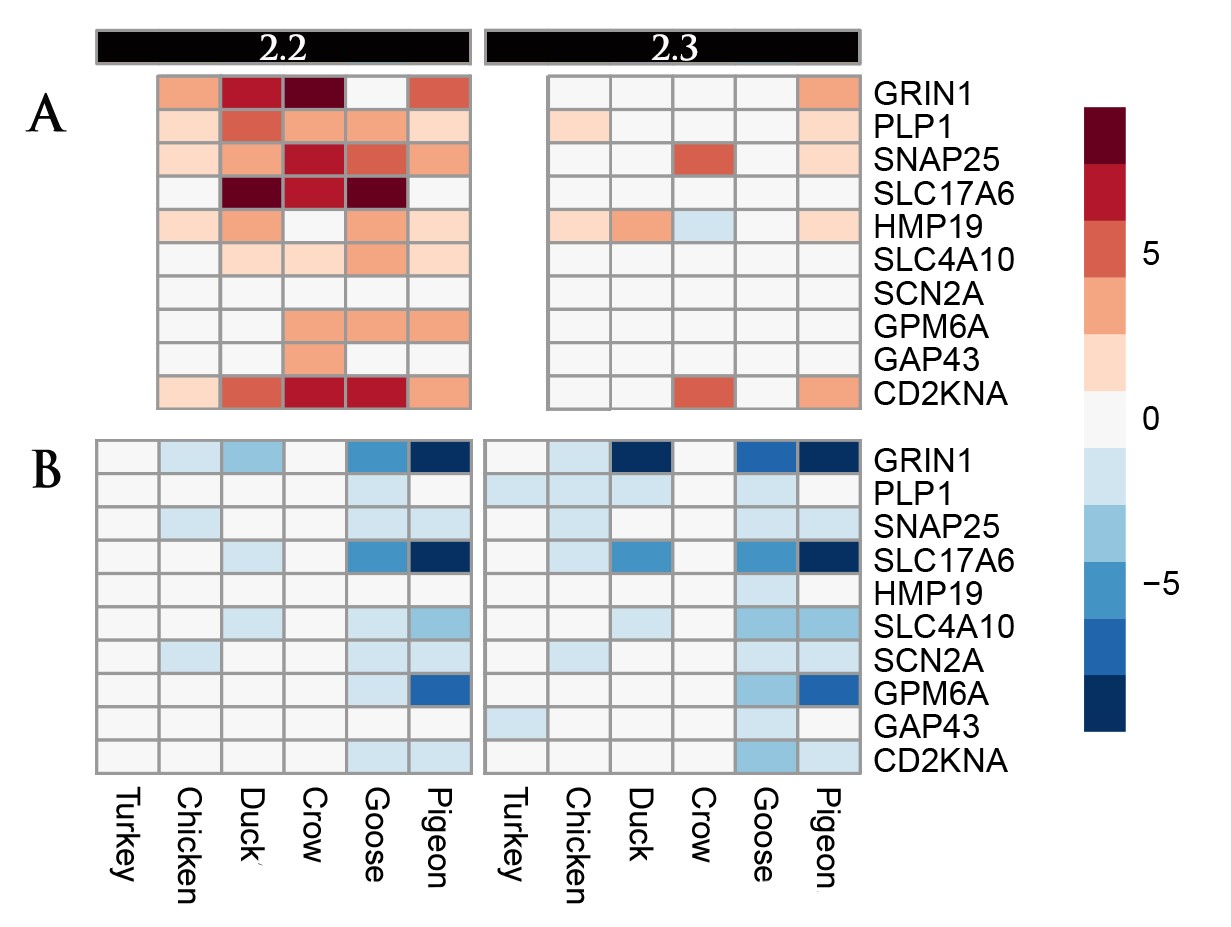


**Figure S4:** Heat map diagram of differential expression of select nerve function genes at 48 h in (**A).** Lung and (**B).** Ileum. Legend is logFC relative to control samples.


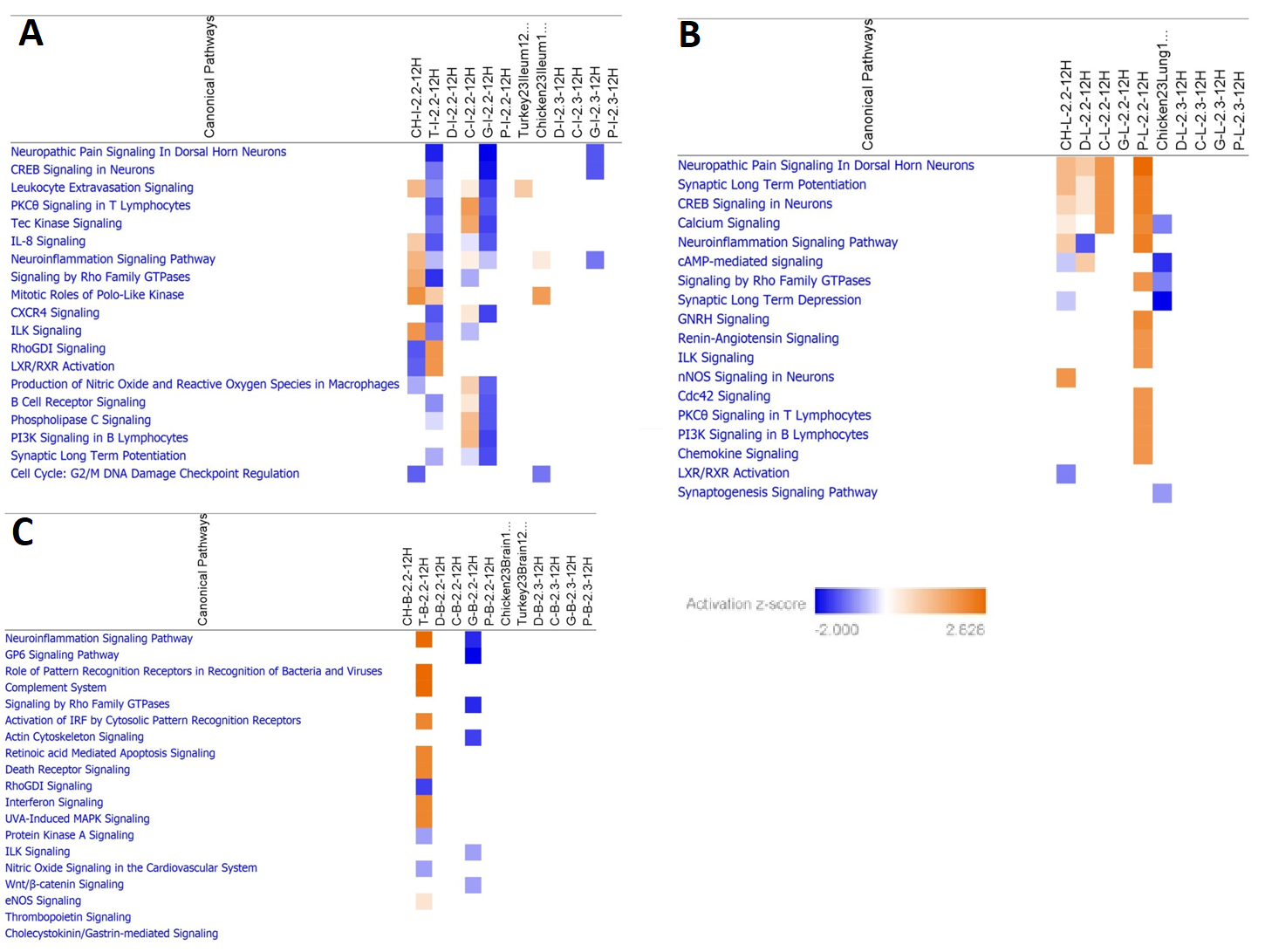


**Figure S5:** Pathway comparison at 12 h in ileum (A), lung (B) and brain (C).


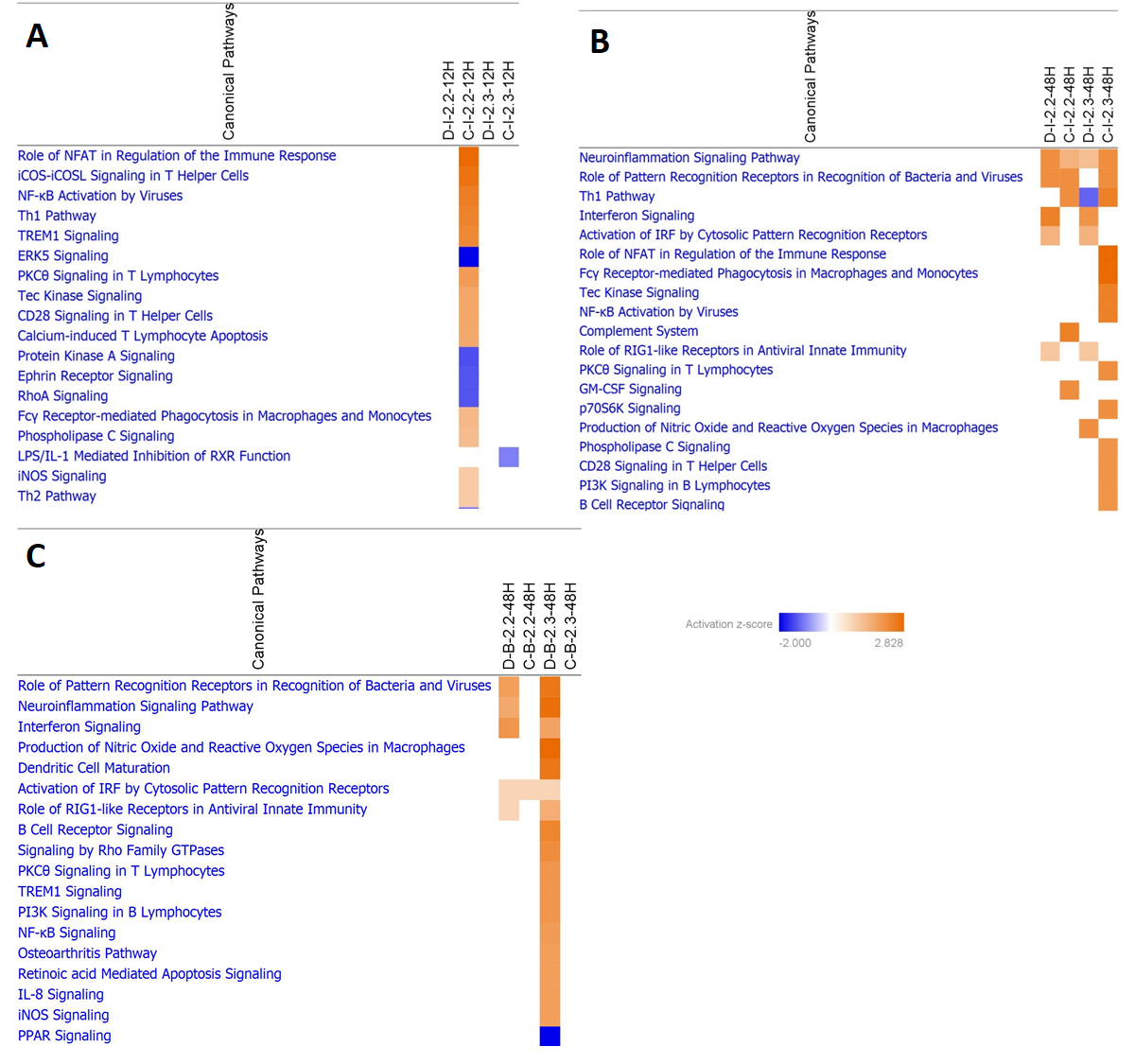


**Figure S6:** Pathway analysis of crow and duck in ileum at 12 h (A) and 48 h (B) and brain at 48 h (C). No significant pathway enrichment was found in brain at 12 h.


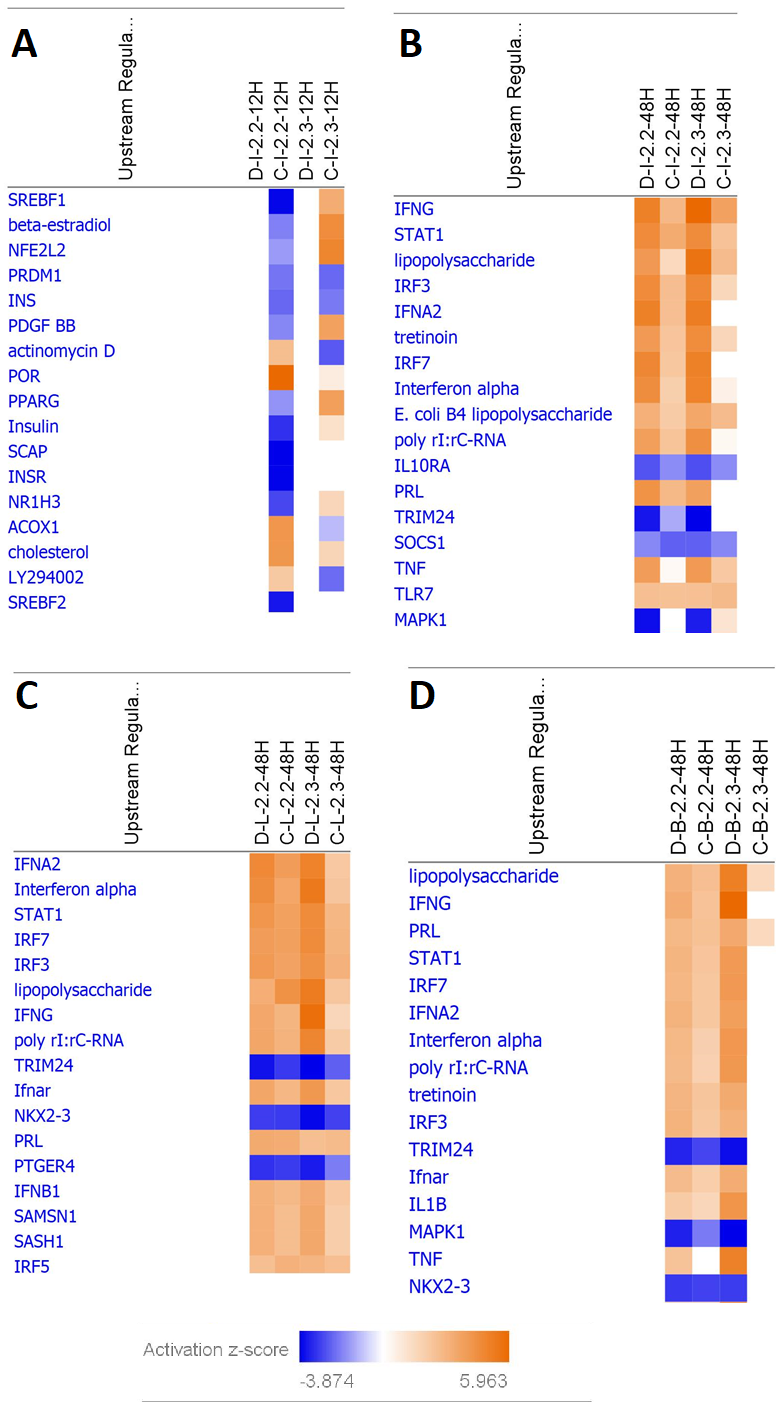


**Figure S7:** Upstream regulator enrichment in ileum at 12 h (A), 48 h (B), lung at 12 h (C) and brain at 48 h (D).


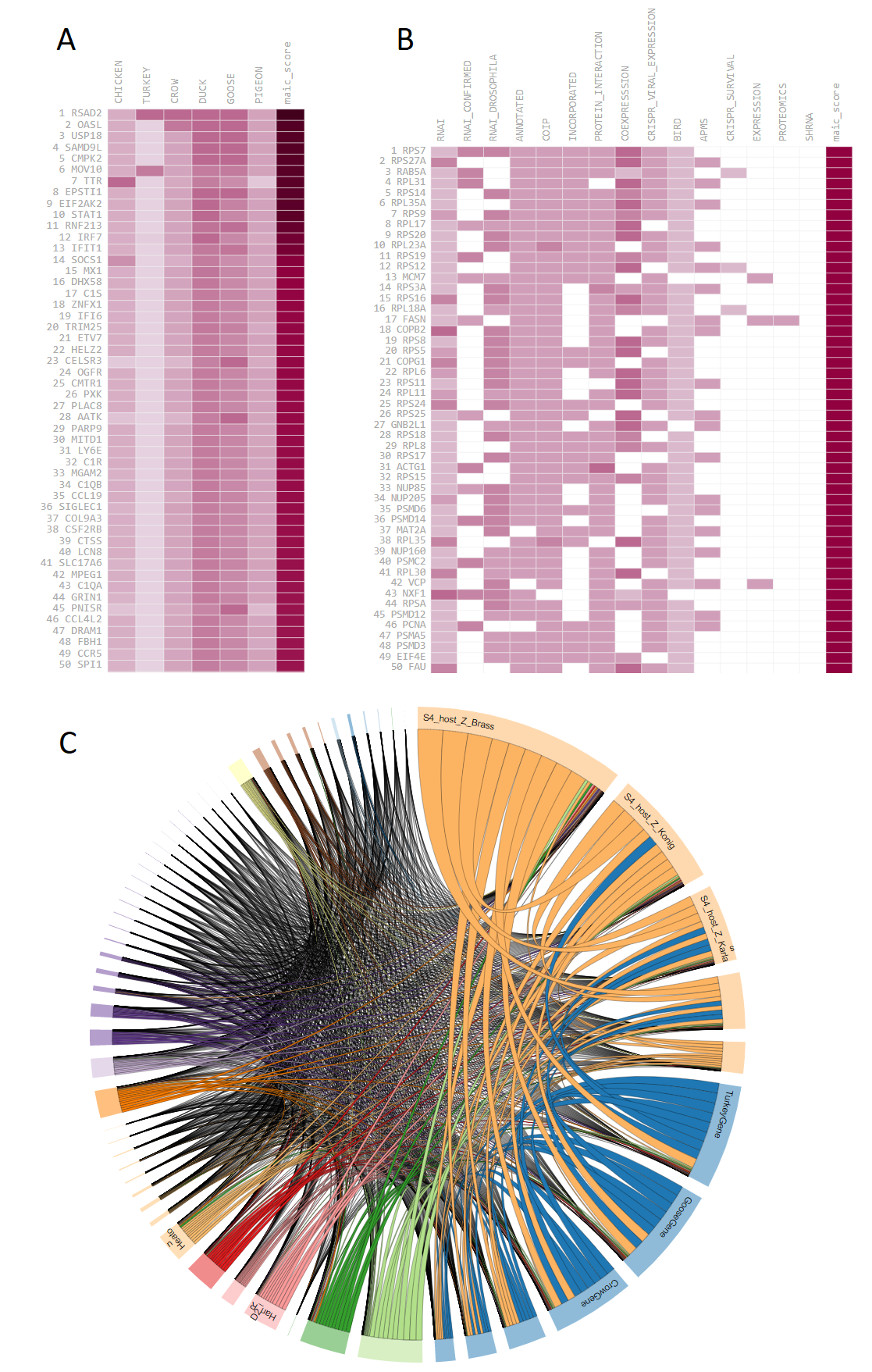


**Figure S8:** MAIC results for host response gene. A - DEGs from each bird species were pooled from tissues and timepoint, and ranked according to FDR, and a heatmap including the top ranked genes based on shared information content constructed. B – Ranked lists from figure A were included in a MAIC analysis with a range of data sources for human influenza host factors, and a heatmap including the top ranked genes based on shared information content constructed. C – A graph representing the shared information content from each data source after MAIC; size of data source blocks is proportional to the information content. Lines showing shared information content are coloured according to the dominant data source.
